# Global distribution, traditional and modern detection, diagnostic, and management approaches of *Rhizoctonia solani* associated with legume crops

**DOI:** 10.3389/fmicb.2022.1091288

**Published:** 2023-02-06

**Authors:** Muhammad Abdullah Akber, Mustansar Mubeen, Muhammad Aamir Sohail, Sher Wali Khan, Manoj Kumar Solanki, Rida Khalid, Aqleem Abbas, Praveen Kumar Divvela, Lei Zhou

**Affiliations:** ^1^State Key Laboratory for Managing Biotic and Chemical Threats to the Quality and Safety of Agro-products, Institute of Agro-product Safety and Nutrition, Zhejiang Academy of Agricultural Sciences, Hangzhou, China; ^2^State Key Laboratory of Grassland Agroecosystems, Key Laboratory of Grassland Livestock Industry Innovation, Ministry of Agriculture and Rural Affairs, College of Pastoral Agriculture Science and Technology, Lanzhou University, Lanzhou, China; ^3^Department of Plant Pathology, College of Agriculture, University of Sargodha, Sargodha, Pakistan; ^4^Department of Plant Pathology, College of Plant Science and Technology, Huazhong Agricultural University, Wuhan, China; ^5^Department of Plant Science, Karakoram International University, Gilgit, Pakistan; ^6^Plant Cytogenetics and Molecular Biology Group, Institute of Biology, Biotechnology and Environmental Protection, Faculty of Natural Sciences, the University of Silesia in Katowice, Katowice, Poland; ^7^School of Biological Sciences, University of the Punjab, Lahore, Pakistan; ^8^Contec Global Agro Limited, Abuja, Nigeria

**Keywords:** legumes, *Rhizoctonia solani*, distribution, detection, diagnosis, management

## Abstract

Sustainable development relies heavily on a food system that is both safe and secure. Several approaches may lead to sustainability and food safety. An increase in the cultivation of legume crops is one of the approaches for enhancing agricultural viability and ensuring adequate food supply. Legumes may increase daily intake of fiber, folate, and protein as substitutes for meat and dairy. They are also crucial in various intercropping systems worldwide. However, legume production has been hampered by *Rhizoctonia solani* due to its destructive lifestyle. *R. solani* causes blights, damping off, and rotting diseases in legume crops. Our knowledge of the global distribution of *R. solani* associated with legume crops (alfalfa, soybean, chickpea, pea, lentil, common bean, and peanut), detection, diagnosis, and management of legume crops diseases caused by *R. solani* is limited. Traditional approaches rely on the incubation of *R. solani*, visual examination of symptoms on host legume crops, and microscopy identification. However, these approaches are time-consuming, require technical expertise, fail to detect a minimal amount of inoculum, and are unreliable. Biochemical and molecular-based approaches have been used with great success recently because of their excellent sensitivity and specificity. Along with conventional PCR, nested PCR, multiplex PCR, real-time PCR, magnetic-capture hybridization PCR, and loop-mediated isothermal amplification have been widely used to detect and diagnose *R. solani*. In the future, Next-generation sequencing will likely be used to a greater extent to detect *R. solani*. This review outlines global distribution, survival, infection and disease cycle, traditional, biochemical, molecular, and next-generation sequencing detection and diagnostic approaches, and an overview of the resistant resources and other management strategies to cope with *R. solani*.

## 1. Introduction

Legumes include plants in the family *Fabaceae*. Grain and forage legumes are grown on about 180 million ha of the world’s arable land. Grain legumes can provide 33% of human dietary protein nitrogen (N; Mueller-Harvey et al., 2019). More than 35% of the world’s processed vegetable oil comes from legumes, which are excellent dietary protein sources for the poultry and cattle industries. For millennia, forage legumes have formed the backbone of the dairy and meat industries (Willett et al., 2019). They may provide much protein, fiber, and calories if cared for correctly. Forage legumes are necessary even in intensive livestock and milk production when grain crops are primary feed sources to ensure animal health (Rehman et al., 2019). Forage legumes are almost indispensable to emerging nations’ meat and dairy industries. For sustained meat and dairy production on poor and nutrient-deficient soils, forage legumes such as alfalfa and clovers are essential. Food security issues, pressures on the land, and increasing soil degradation have led to increasing research interest in tree legumes in forestry. Livestock readily consumes legume tree fodder because of its high crude protein, mineral, and, in some cases, good digestibility levels. The N advantages of tree fallows may typically be maximized using rock phosphate as a fertilizer. N may also be supplied to an intercropped crop by “alley cropping, “in which crops are cultivated between hedgerows and through tree pruning as mulch or green manure. In the majority of ecosystems, nitrogen is the limiting factor in plant growth (Liu et al., 2017). Legumes may help colonize damaged habitats through their symbiotic qualities, especially those prone to fire (Conti et al., 2021). The formation of root nodules and the subsequent symbiotic N_2_ fixation by suitable rhizobia is a defining characteristic of legumes. Agriculturally significant legumes fix between 40 and 60 million metric tons of N_2_ annually, while legumes in their natural habitats fix an additional 3 to 5 million metric tons (Graham and Vance, 2003). Considering how little nitrogenase is used, this is an impressive feat of efficiency (Boqvist et al., 2018). They also improve soil structure and reduce erosion (Mahmud et al., 2020). Biodegradable polymers, oils, gums, dyes, and inks are only some industrial uses for legumes (Abd El-Naby Zeinab et al., 2014). In traditional medicine, legumes are often employed. Soy isoflavones, along with those found in other legumes, have been lately proposed to do double duty by reducing cancer risks and blood cholesterol. Besides being utilized as food and forage, legumes may be processed into flour and used to bake goods, including bread, doughnuts, tortillas, chips, spreads, and extruded snacks (Assunção et al., 2011). Soybean candies, pop beans, and licorice are all creative applications of legumes (Conti et al., 2021). An enormous challenge for agricultural sustainability is posed by the rapid rise in the global population over the last decade, which is predicted to reach roughly 9.5 billion by 2050, with emerging nations accounting for the majority of this increase (United Nations, 2017). Plant growth, productivity, and nutritional value are all adversely impacted by environmental pressures associated with climate change, compounded by the growing demand placed on arable land and natural resources due to rising populations. Moreover, soil and water quality deteriorate due to the overuse of fertilizers and pesticides. Human health suffers as a result of all these difficulties, with malnutrition rates rising not only in underdeveloped nations but even in wealthy ones, where food is readily accessible but of low quality (Willett et al., 2019). Animal products, such as meat and dairy, have far more severe environmental impacts than plant-based diets (Liu et al., 2021a). Inadequate food supplies, wealthy or unsafe varieties, or a lack of money to buy food pose a threat to the world’s population (Boqvist et al., 2018). Those with less money to spend on food are more likely to be hungry or malnourished due to food insecurity. Food insecurity and famines directly result from social instability and conflict (Vågsholm et al., 2020). As a result, food insecurity is a major risk to society’s well-being and economic and political security (Röös et al., 2022). Safe and secure food production is an integral part of sustainable development. Sustainability and food security may be achieved in several ways. One way to improve sustainability and food security is to increase the production of legume crops (Poore and Nemecek, 2018).

However, legume production has been difficult due to relatively low yields, mainly caused by phytopathogens. Phytopathogens, also known as plant pathogens, cause severe legume diseases, which cause the legumes plants to develop abnormally. Without regard for the specifics of the crop, the soil, or the phytopathogen, contemporary agriculture routinely employs heavy doses of fertilizers and pesticides to combat these hazardous organisms (Abd-Elmagid et al., 2020). The end consequence is less fertile land, environmental deterioration from the overuse of chemicals, and the emergence of pathogens resistant to pesticides and other chemical treatments (Tilman et al., 2002; López Cardona and Castaño Zapata, 2012).

*Rhizoctonia solani* JG Kühn [Teleomorph: *Thanatephorus cucumeris* (AB Frank) Donk] is one of the crucial soil-borne necrotrophic phytopathogens that causes hypocotyl, crown, root, stem collar, bud, and fruit rots, blights, wire stem and damping-off in legume production regions worldwide. It has been estimated that, on average, 20% of annual legume yield loss is due to the *R. solani*, and even in some rare scenarios, 30–60% to complete loss of the legume crops has also been observed (Ajayi-Oyetunde and Bradley, 2018). Furthermore, because of its facultative parasitic ability, it can survive as a saprotroph in the soil. *R. solani* can spread by rain-splashed sclerotia, infested soil debris, and mycelial bridges between plants and infected seeds. While the teleomorph (sexual) stage of *R. solani* allows airborne basidiospore transmission, this is not typical in the fields. The sclerotia, the asexual stage of *R. solani* can remain viable in the soil for several years. Based on hyphal fusion, culture morphology, pathogenicity or virulence, and DNA homology, *R. solani* is a species complex and has been divided into 13 somatically incompatible groups, otherwise termed anastomosis groups (AGs; Abbas et al., 2022a). Hyphal fusion, referred to as anastomosis, occurs only between different AGs isolates. AGs are further subdivided into subgroups based on anastomosis frequency, physiological and morphological characteristics, pathogenic characteristics, and biomolecular, biochemical, genetic, and DNA homology characteristics. Within an AG, isolates could have similar symptoms and host preferences. For example, legume blights are caused by some of these AGs, whereas other AGs are responsible for roots, stems, and seed rots (Harikrishnan and Yang, 2004). Adaptation to diverse ecological regions causes AGs variety, although the geographic dominance of certain AGs types is poorly understood. The genetic diversity of *R. solani* has allowed it to thrive in a wide variety of habitats and hosts (Godoy-Lutz et al., 2008; Gónzalez et al., 2016). Traditional, biochemical and molecular approaches are used to detect and diagnose *R. solani* (Oladzad et al., 2019). Traditional approaches include visual examinations of symptoms on host legume crops, isolation, and incubation of *R. solani* using an appropriate growth medium, morphology, and microscopy identification. The difficulty of identifying and classifying *R. solani* through traditional approaches stems from the fact that cultural conditions modify morphological characteristics. Further, traditional approaches are non-specific and less reliable and could not detect low inoculum and latent infections of *R. solani*. In contrast, biochemical and molecular-based approaches detect and diagnose *R. solani* efficiently due to their high sensitivity and specificity. In recent years, next-generation sequencing (NGS) has been used for detecting and diagnosing *R. solani* (Das et al., 2016; Patil and Solanki, 2016). With new knowledge of detecting and diagnosing *R. solani*, the dissemination and spread of infection of *R. solani* will be controlled, and consequently, the economy of the farming community will be improved (Das et al., 2016; Patil and Solanki, 2016). The following parts have been included in the review: (1) the global distribution of *R. solani* on legumes (alfalfa, soybean, chickpea, pea, common beans, and peanuts), (2) legume diseases caused by *R. solani*, (3) survival, infection and disease cycle, (4) traditional, biochemical, molecular, genomic, transcriptomic and NGS diagnostic approaches for *R. solani*, and (5) disease management approaches and resistance sources.

## 2. Global distribution of *Rhizoctonia solani* on legume crops

**Figure 1:**
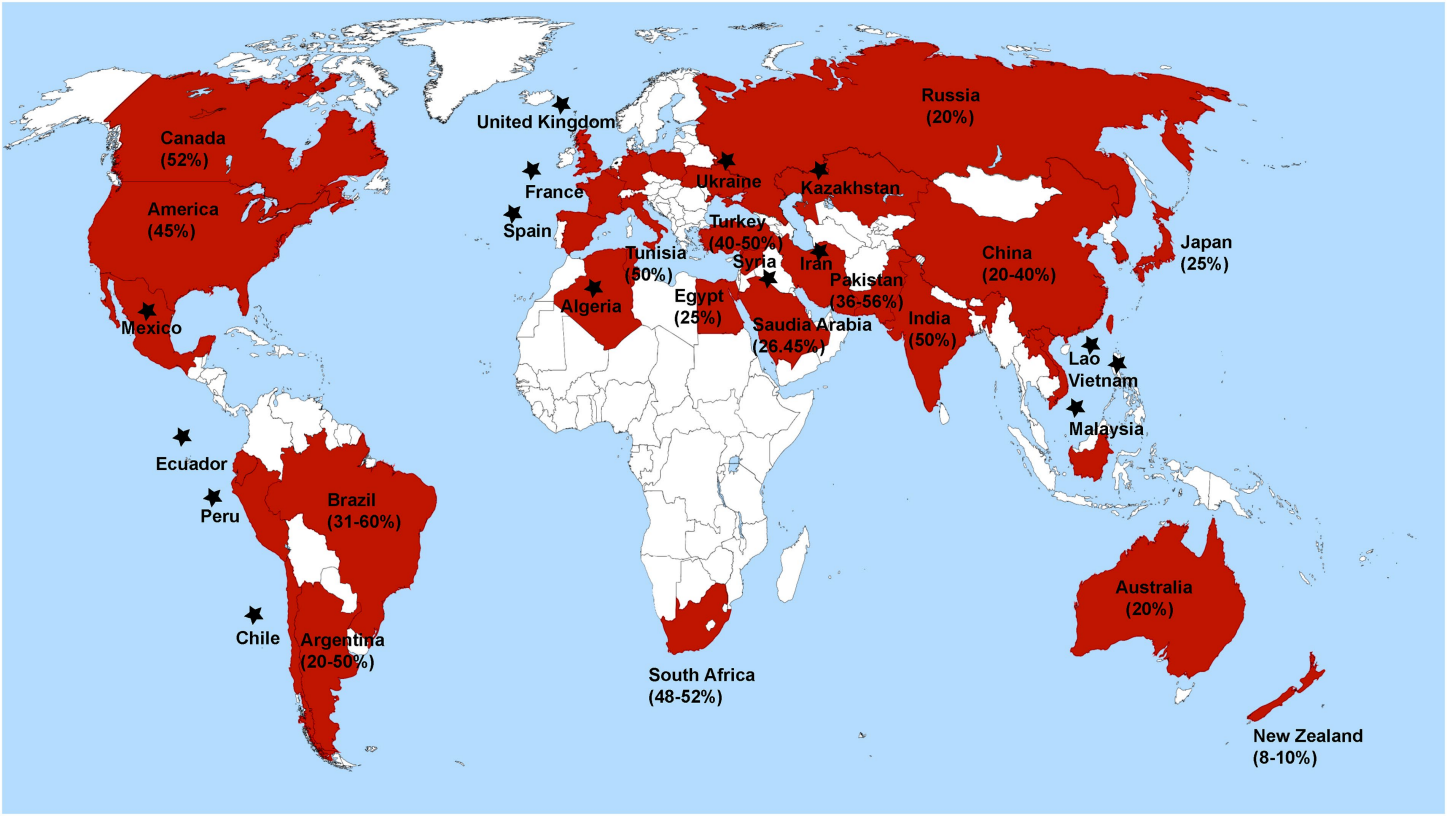
Global distribution and percent disease severity of *R. solani* associated with leguminous crops. Star has been placed over countries where *R. solani* is present, but disease severity has not been documented.

**Figure 2:**
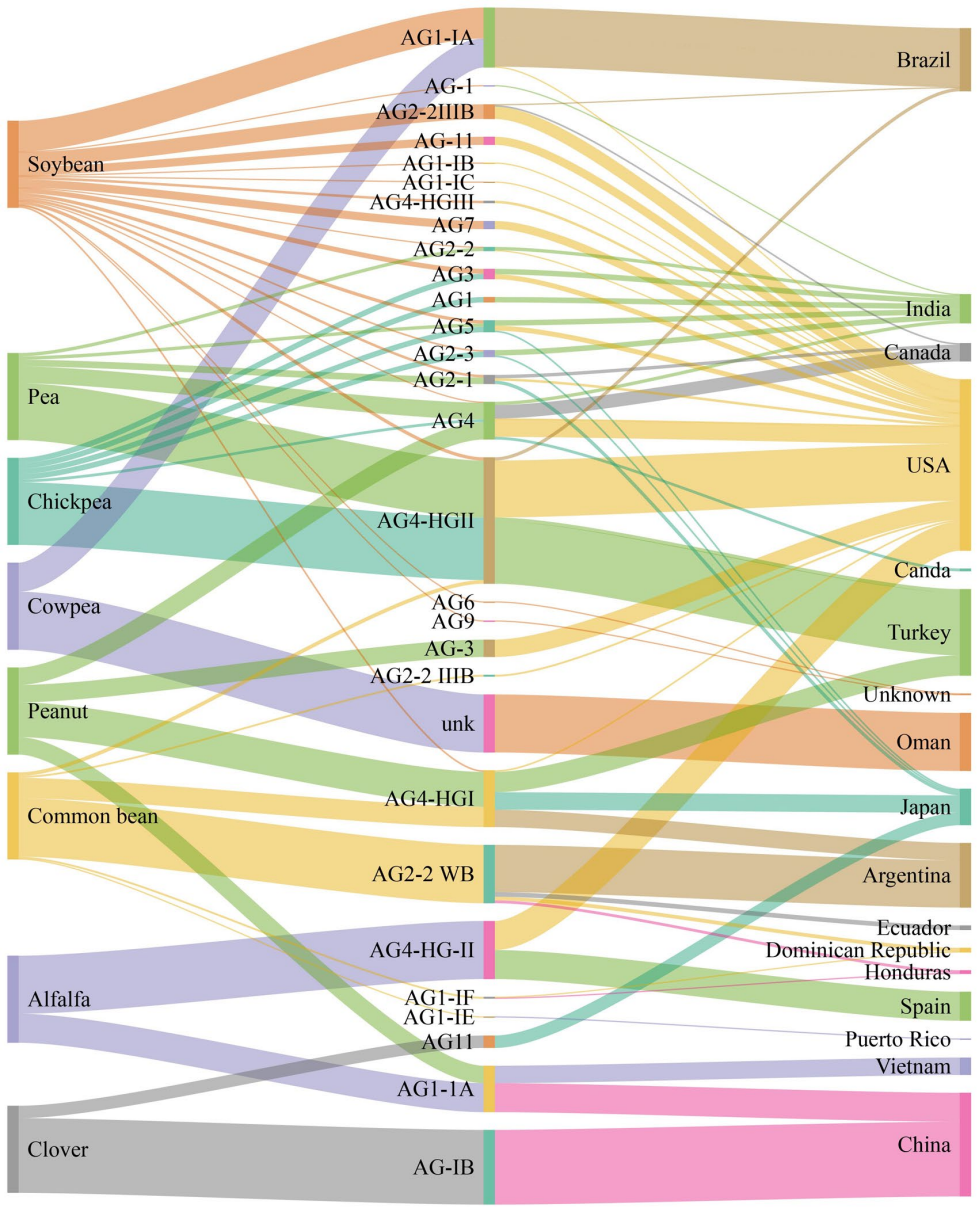
Number of anastomosis groups (AGs) and their subgroups of *R. solani* associated with legumes crops and geographical origins.

*Rhizoctonia solani* causes severe diseases such as blights (web and foliar), damping-off, and rots (seed, stem, root, crown, collar, and hypocotyl) in alfalfa, soybean, chickpea, pea, common beans, and peanuts (Basbagci and Dolar, 2020). The geographical distribution and disease severity of *R. solani* associated with legume crops are shown in Figure 1 (Abbas et al., 2022b). *R. solani* is causing severe yield losses and various diseases in legume crops in different countries, as shown in Table 1. Furthermore, *R. solani* is a species complex and has been classified as 13 AGs based on hyphal anastomosis reactions, morphology, pathogenicity/virulence, and DNA homology (Salazar et al., 2000; Gonzalez et al., 2001; Bai et al., 2014; Dubey et al., 2014; Gónzalez et al., 2016; Spedaletti et al., 2016; Basbagci and Dolar, 2020). AG 1, 2, 3, and 4 are present worldwide, while the occurrence of remaining AGs varies as AG-5; Canada, Germany, Israel, and Japan, AG-7; Japan, AG-8; Australia, AG-9; America and Canada, AG-BI; Japan, are widely known. AG-6, AG-7, AG-8, AG-9, and AG-B1 have been identified recently (Ogoshi, 1987, 1996). Among these AGs, AG-1, AG-2, AG-3, AG-4, and AG-5 are frequently reported from legume crops, as shown in Figure 2 (Hane et al., 2014).

**Table 1:** Legume crops diseases caused by anastomosis groups of *R. solani* across different countries.

Legume	Disease	AG group	Yield loss/Not documented	Country	References
Alfalfa	Damping-off	*R. solani*	26.45%	Saudi Arabia	Al-Askar et al. (2013)
	Damping-off	*R. solani*	25%	Egypt	Abd El-Naby Zeinab et al. (2014)
	Root rot	*R. solani*	20–40%	China	Cong et al. (2016)
	Seed rot	*R. solani*	Not documented	USA	Berg et al. (2017)
	Damping-off				
	Root rot	*R. solani*	0.0–60.0%	Egypt	El-Garhy and El-Wakil (2014)
	Damping-off				
	Root rot	*R. solani*	Not documented	Saudi Arabia	El-Meleigi et al. (2017)
	Damping-off				
	Stem rot	AG-2-IIB	Not documented	Japan	Misawa and Kurose (2019)
	Root rot	*R. solani*	Not documented	USA	Samac et al. (2015)
	Damping-off				
	Damping-off	*R. solani*	Not documented	Italy	Bonanomi et al. (2011)
	Root disease	*R. solani*	Not documented	Saudi Arabia	Alsohim (2020)
Soybean	Foliar blight	AG-1-IA	Not documented	USA, India, Brazil	Liu et al. (2017)
	Root disease	AG-4	Not documented	Turkey	Erper et al. (2011)
	Seedling disease	AG-2-2IIIB	Not documented	USA	Zhao et al. (2005)
		AG-4			
		AG-5			
	Seedling blight	*R. solani*	Not documented	China	Lu et al. (2015)
	Damping-off,	AG-2-2IIIB	Not documented	USA, Canada	Ajayi-Oyetunde and Bradley (2017)
	Root rot				
	Hypocotyl rot				
	Seedling blight	*R. solani*	52%	Canada	Chang et al. (2017)
	Root rot				
	Root rot	AG-2-2 AG-4	45%	USA	Muyolo et al. (1993)
		AG-5			
	Damping-off	*R. solani*	31–60%	Brazil	Chela Fenille et al. (2002)
	Root rot				
	Hypocotyl rot				
	Foliar disease	AG-1-IA	Not documented	Rondônia	Chavarro-Mesa et al. (2020)
	Aerial blight	AG-2	Not documented	USA	Spurlock et al. (2016)
	Sheath blight				
	Root rot	*R. solani*	Not documented	Egypt	Amani et al. (2017)
Chickpea	Root and collar rot	AG-2	50%	Tunisia	Youssef et al. (2010)
	Damping-off	AG-4	Not documented	Turkey	Basbagci and Dolar (2020)
	Root rot	AG-5			
	Wet root rot	AG-3	Not documented	India	Dubey et al. (2011)
		AG-5			
	Seedling blight	AG-4	Not documented	Canadian Prairies	Hwang et al. (2003)
	Root rot				
	Root rot	AG-5	3.4	Eastern Anatolian region	Demirci et al. (1998)
	Seedling disease	AG-5	Not documented	Egypt	Maurice et al. (2010)
	Wet root rot	*R. solani*	Not documented	India	Prasad et al. (2014)
	Wet root rot	AG-3	50%	India	Ganeshamoorthi and Dubey (2013)
		AG-5			
		AG-1			
		AG-2			
		AG-4			
	Root diseases	*R. solani*	Not documented	Egypt	Abdel-Monaim (2011)
	Root rot	*R. solani*	Not documented	Ethiopia	Yimer et al. (2018)
Pea	Root rot	AG-4	Not documented	Saudi Arabia	Al-Askar and Rashad (2010)
	Seedling blight	AG-4	Not documented	Canada	Hwang et al. (2007)
		AG 2–1			
	Root Rot,	AG-4	75%	USA	Sharma-Poudyal et al. (2015)
	Stunting	AG-2-1			
	Seedling disease	AG-8			
	Root rot	AG-4-(HG)-II	Not documented	USA	Mathew et al. (2012)
		AG-5			
	Root rot	AG-1-IA	Not documented	China	Yang et al. (2005)
	Stem rot	AG-4-HG-II	Not documented	USA	Woodhall et al. (2019)
	Damping off,	AG-2-1, AG-2-2, AG-4,	31%	Canada	Woodhall et al. (2019)
	Crown rot	AG-5,			
		AG-9,			
		AG-11			

## 3. Diseases of legumes caused by *Rhizoctonia solani*

*Rhizoctonia solani* has a destructive lifestyle with a vast host range. Worldwide different economically important crops are affected by this fungus. *R. solani* attacks members of *Poaceae*, *Rubiaceae*, *Fabaceae*, *Solanaceae*, *Moraceae*, *Malvaceae*, *Linaceae*, *Araceae*, and *Amaranthaceae* families. Disease symptoms on different hosts are stem rot and canker, hypocotyl rot, black scurf, pod rot, blights, root rot, crown rot, pre, and post-emergence damping-off (Patil and Solanki, 2016). Among the legumes, soybean (*Glycine max*) is affected by *R. solani*, which causes aerial and underground diseases. *R. solani* can survive and infect soybean with a wide range of temperatures and moisture levels (Dorrance et al., 2003). Moreover, seed decay and pre-emergence damping-off lead to missing stand, and replanting such fields may be required if the attack is severe (Bradley et al., 2002). Soybean yield production is affected on a small scale because it can tolerate decreased stands. However, the yield can be reduced with the age of the plant because older plants cannot handle missing stands. In some cases, soybean yield was reduced by up to 48% by the impact of seedling diseases caused by *R. solani* (Koenning and Wrather, 2010). Chickpea (*Cicer arietinum*) is one of the essential cool season leguminous crops cultivated in dry areas. It is primarily grown in drylands. The crop is affected by severe diseases throughout its life cycle (Nene et al., 2012). It is reported that more than 50 plant pathogens attacked chickpea worldwide. However, the major fungal diseases are *Fusarium* wilt, *Ascochyta* blight, and root rot caused by *R. solani* (Bayraktar and Dolar, 2009). *R. solani* causes root rot and collar rot which can be seen when moisture is high at every stage of plant growth (Prasad et al., 2014). Pea (*Pisum sativum*) is a leguminous crop used as a vegetable or dry pea. Worldwide, several soil-borne pathogens cause seed rot, pea root rot, and damping-off (Heyman et al., 2013). *R. solani* causes serious pea diseases such as seedling blight, damping-off, and root rot (Sharma-Poudyal et al., 2015). Pea root rot is caused by various AGs of *R. solani* (Mathew et al., 2012). However, AG-4 is mainly responsible for causing pea root rot worldwide (Kraft and Pfleger, 2001). Alfalfa (*Medicago sativa*) is one of the oldest forage legume crops cultivated worldwide. Different soil-borne pathogens attack the alfalfa crop, resulting in poor stand establishment, decreasing its yield, forage quality, and longevity*. R. solani* is also known to infect the crown at the place where new buds begin (Eken and Demirci, 2003). Lentil (*Lens esculinta*) is one of the oldest leguminous crops; its protein content ranges from 22 to 35 percent (Stodart et al., 2007). *R. solani* causes damping-off disease in lentils (Duarte et al., 2018). It is the main disease responsible for high yield reduction and sometimes total yield loss of lentils. It is found that damping-off caused by *R. solani* is decreased in lentils when it is planted after maize and rice and increased when planted after soybean and cotton (Hamdi et al., 2002). Common bean (*Phaseolus vulgaris*) is the third-largest leguminous crop after soybean and peanut. *R. solani* infects common bean plants through root rot (Valentín Torres et al., 2016). Moreover, *R. solani* can attack common bean at any age, and pathogens enter *via* wounds or by forming infection cushions and penetrating the epidermis (Guerrero-González et al., 2011). Peanut (*Arachis hypogaea*) is cultivated worldwide as a food and oilseed crop. Peanuts are affected mainly by soil-borne pathogens because their pods are produced below ground. Soil-borne pathogens such as *R. solani* are not easy to control because fungicides spray cannot disperse completely through the peanut canopy (Guerrero-González et al., 2011). *R. solani* infects peanuts and causes damping-off, seed decay, pod root, root rot, and limb rot (Marsalis et al., 2009). Also, AG-4 is the most virulent isolate to cause diseases in peanuts (Thiessen and Woodward, 2012).

## 4. Survival, infection, and disease cycle

As aforementioned, seed decay, pre- and post-emerging damping off, rots (hypocotyl, crown, stem, collar, and root), stem canker, chlorosis, stunting, and blights (web and foliar) are well-established legume crop diseases. Different AGs of *R. solani*, have been associated with these diseases. These diseases’ occurrence, severity, and prevalence are affected by primary infection sources, management practices, environmental factors, and cultivars’ ability to resist diseases. Among the environmental factors, temperature and moisture play a significant role in the severity, occurrence and prevalence of legume diseases. Cool soil conditions slow seedling growth and emergence, making emerging plants susceptible to *R. solani*. Seed decay and pre-emergence damping off are more common in fields with high inoculum amounts or cool, wet weather. Lower inoculum pressure rots plant roots or hypocotyls. Unlike other legume crop pathogens, *R. solani* can infect legumes of varying temperatures and moisture levels. For example, *R. solani*, AG-2-2IIIB, can thrive and infect soybeans at temperatures as high as 35°C (Ajayi-Oyetunde and Bradley, 2018). Similarly, severe root rotting of alfalfa, caused by *R. solani* occurred at 65% water holding capacity and less at 45%. Likewise, each anastomosis group (AGs) of *R. solani* requires a certain temperature to cause legume disease. *R. solani* also infects legumes at cold temperatures (15–18°C), which slows down the growth of their seedlings (Abbas et al., 2022b). It is common for fields to need to be replanted if there are missing stands due to seed decay or pre-emergence damping off. Once plants have emerged, they are susceptible to hypocotyl and root rot, which may or may not result in death. Plants exhibiting symptoms often have a reddish-brown discoloration in the cortical layer of the lateral roots or the lower part of the stem near the soil line. If a plant’s roots are afflicted, the plant may not develop as vigorously and cannot absorb as much water and nutrients. In addition to having a chlorotic and stunted appearance, plants with less access to soil water may ultimately wilt and die. Generally, a plant’s resistance improves as it ages, making older plants less vulnerable to diseases. However, older plants may be more vulnerable to infection and mortality if exposed to unfavorable environmental conditions (Ajayi-Oyetunde and Bradley, 2018).

**Figure 3:**
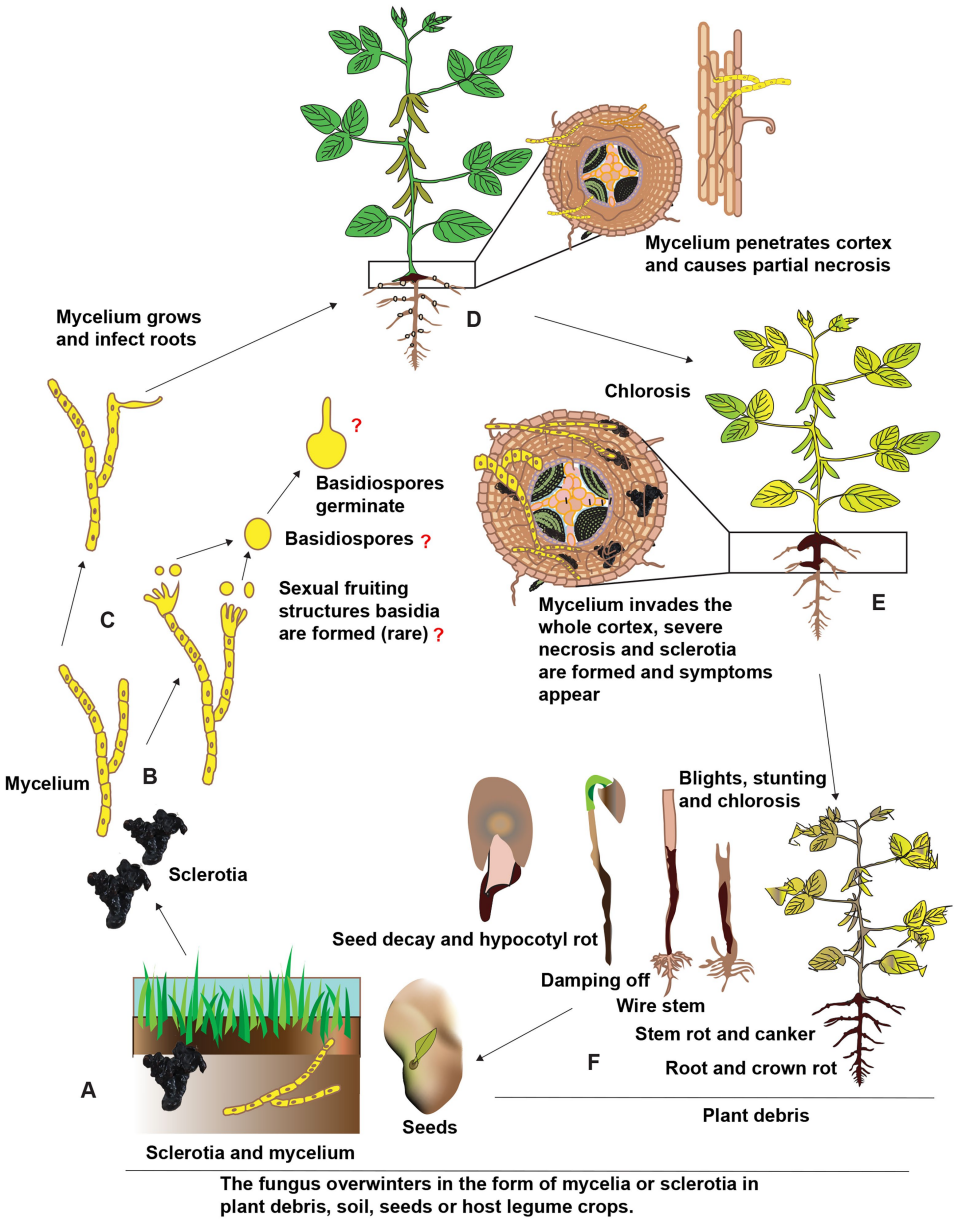
Schematic representation of the presumed disease cycle of *R. solani* on legume crops. **(A)** The fungus overwinters in the soil as sclerotia and mycelium as well as in plant debris and seeds in the form of mycelium. **(B,C)** Under favorable environmental conditions, the immature hyphae germinate and grow, although sexual fruiting structures like basidia and basidiospores are uncommon. **(D)** The mycelium penetrates roots near the soil line and is colonized in inter and intracellular spaces. **(E)** The mycelium proliferates further in the cortex ultimately resulting in necrosis finally, sclerotia are formed in and on infected tissues and different symptoms appear on stems and roots of different legume crops. **(F)** Above-ground symptoms include chlorosis, blights, stunting, and finally death, the fungus also infects seeds and seedlings causing seed decay, damping-off, and other symptoms. Reconstructed from Abbas et al. (2022b).

*Rhizoctonia solani* is an excellent competitor with other soil-dwelling saprophytes despite being a facultative parasite. Long-lasting “nutrient-independent propagules” (sclerotia) help it persist in the soil. However, it is unclear whether or not basidiospores have a function as an inoculum source for legume diseases. Undifferentiated hyphae or monilioid cells give birth to sclerotia, which then germinate into mycelia and act as an inoculum source for infection and disease dissemination. Rhizoctonia infections typically begin with the sclerotia germinating to produce mycelia and then make their way toward the legume hosts. In response to legume crops exudates, the fungus forms a mycelium, which then gives rise to hyphal attachment, hyphal growth along the host’s epidermal cell walls, the formation of T-shaped branches with appressoria-like infection structures (Abbas et al., 2022a). Sclerotia can germinate and develop hyphae, invade the root cortex, and persistently spread inside and on the surface of roots under favorable environmental conditions. This causes the roots to develop dark streaks down their length, and eventually, the roots will rot and die. The top of the root head also becomes a deep shade of brown or perhaps black. Symptoms on the foliage might manifest as chlorosis, blights, stunting, or wilting of the leaves. Seeds infected with *R. solani* are unlikely to germinate; even if they do, the resulting seedlings will likely die shortly after or shortly after emergence. Recurring sclerotia arises, completing the disease cycle as shown in Figure 3, and may survive for years under extreme environments such as extreme heat or cold, prolonged periods without food or water, the presence of harsh chemicals, or high levels of radiation (Abbas et al., 2022b).

## 5. Detection and diagnostic approaches for *Rhizoctonia solani* in legumes

Various techniques for detecting fungal pathogens in their respective host plants are discussed in the literature (Ahmad et al., 2019). Some basic traditional and biochemical techniques are used to detect *R. solani* and diagnose the diseases caused by *Rhizoctonia* species, particularly in legumes are shown Table 2. In contrast, molecular, transcriptomic, next generation sequencing approaches are implied for some advanced diagnoses (Table 2). A few of these techniques are listed below.

**Table 2:** Advantages and disadvantages of different detection and diagnostic approaches for *R. solani* associated with legume crops.

Diagnostic methods	Assays/Platforms	Advantages	Disadvantages
Traditional approaches	Visual examination	Symptom-based	Symptoms common to many pathogens
		Cheaper	Not suitable for latent infection
	Incubation methods	Good for high-incidence fungi	
		Providing information about the viability	
		Cheaper	Fungal reproductive structures are not always produced on agar media Ambiguous nature of anastomosis grouping of isolates
		Simplicity of application	Time-consuming
			Require mycological skills
			Low sensitivity
			Not always reliable
			Low specificity
	Microscopy	Right-angle branching of septate hyphae	Cannot differentiate AGs or AGs subgroups of *R. solani*
Biochemical approaches	Fatty acid profiling, and	Helpful in examining genetic diversity among AGs	The lack of specificity of the antibody
	Pectin enzyme analysis		Commonly not used for direct soil or plant material testing
	Isozyme polymorphism		Also, require skilled persons
	Serological methods	Do not require pure isolation of the pathogen	Cannot distinguish AGs subgroups of *R. solani*
		Applicable to *R. solani*, which is a necrotrophic pathogen	
			Lack of species-specific antibodies
			cannot distinguish between pathogenic and non-pathogenic
			Detect non-viable pathogens, which can result in erroneous interpretations
	Matrix-assisted laser desorption/ionization (MALDI)-time of flight (TOF) mass spectrometry (MS)	Analysis of non-volatile high-molecular compounds (peptides, proteins, carbohydrates, oligonucleotides, synthetic polymers, organic complex compounds, etc.) of *R. solani*	Required pure culture of *R. solani*
Polymerase chain reaction (PCR)-based approaches	Conventional PCR	Rapidity, specificity, sensitivity, and easy interpretation	Compounds inhibit DNA amplification, resulting in false negatives
		Distinguish between closely related organisms	Gives only qualitative data
			The presence of low levels of inoculum can be a problem and may result in a false negative
			Cannot distinguish between viable and non-viable inoculum of *R. solani*
	BIO-PCR	Detect fungus at very low levels	
		Highly sensitive PCR technique	
		Elimination of PCR inhibitors	
		Detection of viable cells	
		Avoiding false positives	More expensive than conventional PCR, primarily if selective media are used
	DNA–DNA hybridization assay	DNA relatedness in AGs	Requires the entire genome of the species
			Time-consuming pairwise comparison
	Nested PCR	Detection of a target DNA at several-fold lower levels	More labor intensive
			More costly
			More prone to contamination
	Real-time PCR	Allows quantification of specific DNA targets	
		Reduces the risk of false positives due to cross-contamination of the reaction mixtures	
		Less time-consuming	
		High sensitivity	Issues with sensitivity, repeatability, and specificity
		Use multiple primers to reduce costs and labor	Need appropriate target DNA fragments for the design of the primers and probes is problematic
	SCAR Approach	Amplify members from the same genus	
		High specificity using soil or infected plant parts	Need for sequence data to design the PCR primers
		Quick and easy to use	Require effort and expense in primers designs
		They have high reproducibility and are locus-specific	
	Fingerprinting Techniques	Amplify random tandem repeats on genomic DNA	Necessitate pure fungal cultures and are not ideal for directly exploring plant material, soil, or growing media
		Detect species-specific patterns	
		Phylogenetic structure of different microbial species	
		understanding of population structure	
	Cross-Hybridization using UP-PCR	A single UP-primer is used to determine the sequence similarity (homology) of unknown *Rhizoctonia* strains	Temperature of hybridization and salt concentration
			Concentration of the denaturant in the buffer
			Length and nature of the probe sequence.
Transcriptomic approaches		Closely related strains of *R. solani* with distinct characteristics may be gleaned through comparative sequencing analysis	Need intensive work with massive sequencing data because of the *R. solani* multinucleate nature
Genomic approaches		Draft genome sequence	Extensive host range and virulence
Loop-mediated isothermal amplification (LAMP)		Simple	
		Cost-effective	
		A rapid method for specific detection of genomic DNA	
		All of the reactions can be carried out under isothermal conditions	
		It does not require expensive equipment	
		Fewer preparation steps	Heavy reliance on indirect detection methods like turbidity and non-specific dyes, often leads to the detection of false positive results.
		Highly specific	
		The amplification efficiency of LAMP is exceptionally high	
Next-generation sequencing	Life Sciences 454 sequencing	Analysis of RNAs	
		Rapid identification	Computational resources required for the assembly, annotation, and analysis of sequencing data
		Mycobiome can be studied	Lower per read accuracy
		Taking advantage of PCR emulsion	
		A highly efficient *in vitro* DNA amplification method	
		Can produce 80–120 Mb of sequence in 200- to 300-bp reads in a 4 h run	
	AB/SOLiD technology	Sequencing by oligonucleotide ligation and detection (SOLiD).	Computational resources required for the assembly, annotation, and analysis of sequencing data
		Employs few inputs and is based on chemistry involving the ligation of di-base labeled probes.	Lower per read accuracy
		The typical read length for a SOLiD run is 25–35 bp, and the total amount of sequencing data generated is 3–4 Gb for 5 days	
	Illumina/Solexa sequencing	Similar to the Sanger-based methods	Computational resources required for the assembly, annotation, and analysis of sequencing data
		Solexa terminators are reversible, permitting the continuation of polymerization following fluorophore detection and deactivation	Lower per read accuracy
		Solid-phase amplification involves the immobilization of sheared DNA fragments on a solid surface (flow-cell channel)	
		The average run size is 40–50 Mb (read duration is 50–300 bp)	

References: Ogoshi (1996), Lübeck and Lübeck (2005), Lakshman et al. (2016), Xia et al. (2017), Chavarro-Mesa et al. (2020), Misawa et al. (2020), Chun et al. (2022).

### 5.1. Traditional approaches

Traditional approaches are based on evaluating fungal morphology for genus confirmation, which has been well-documented (Ahmad et al., 2020). These conventional procedures are also used to determine nuclear status for species determination, and the use of AG tester isolates for AGs and AG sub-group determination in the case of *R. solani* in various legumes and microscopy identification such as right-angle branching of septate hyphae (Choudhary et al., 2020). The anastomosis reaction was used to characterize the AGs of the *R. solani* isolates. If isolate hyphae attract and fuse, the isolates belong to the same AG; other AGs do not exhibit this behavior. Other features that helped identify *R. solani* AG and subgroups were thiamine need, optimal growth temperature, sclerotium type, host origin, and symptoms (Ogoshi, 1996). Various detection techniques have been investigated qualitatively and quantitatively, including baiting with susceptible host legume crop (Papavizas and Davey, 1959), wet sieving and direct microscopic examination (Boosalis and Scharen, 1959), incubating immersion tubes in soil (Martinson, 1963)and plate profiling (Andersen and Huber, 1965). These methods only require basic laboratory equipment but are labor-intensive, making them unsuitable for large-scale environmental studies.

Hyphal anastomosis is the standard for classifying and subdividing strains of this sexually cryptic *R. solani* fungus (Sneh et al., 1996). Pathogenicity, colony morphology, DNA complementarity, pectic zymograms, etc., may all be used to distinguish between subgroups within an AG. The *R. solani* AGs are generally thought of as genetically isolated, non-interbreeding populations, with just 0–30% DNA homologies across isolates of different AGs and 5% nucleotide homology between any two AGs. Sequences from the internal transcribed spacer (ITS) and the beta-tubulin gene revealed AG-1, AG-4, AG-6, AG-8, and AG-BI had monophyletic origins, whereas AG-2 has polyphyletic origins (Gónzalez et al., 2016). Although AG-2 and AG-3 are assumed to be homothallic for mating, AG-1, AG-4, and AG-8 are characterized as heterothallic and have outcrossing population dynamics. According to histopathology findings, *R. solani* AGs elicit various host responses, especially regarding pectin breakdown. The susceptibility of various *Rhizoctonia* species and AGs to fungicides may vary. For this reason, determining the genus, species, AGs, and AG subgroups of *R. solani* is essential for creating effective disease control strategies.

#### 5.1.1. Drawbacks of traditional methods

Cultural morphology, including colony color, monilioid cells, sclerotia, and other mycelium characteristics, and the biochemical and/or anastomosis behavior of hyphal fusion reactions of vegetatively compatible isolates with tester isolates are the sole basis for the differentiation, grouping, and identification of *R. solani* strains in the traditional method (Rehman et al., 2019). Isolate anastomosis might range from entirely successful to completely ineffective. Hyphal anastomosis responses have been used to classify *R. solani* strains into different AGs. Differences in culture appearance, morphology, host range, pathogenicity, thiamine needs, and hyphal fusion frequency have led to the subdivision of these AGs. Some of the categories have even more granular subcategories. Even though there are several problems with using anastomosis responses to classify *Rhizoctonia* isolates, this approach has been utilized for a long time. Instability in genetics, the environment, or nutrition may prevent anastomosis between two isolates of the same AGs. Establishing a fusion reaction also takes time and might be hard to decipher (Oladzad et al., 2019).

Due to the availability of bridging isolates, it might be difficult to correctly assign an isolate to the appropriate AGs. Defining “bridging isolates” as the apparent hyphal fusion of specific isolates from one AG with isolates from another AG is straightforward. AG-2, AG-3, AG-6, and AG-11 isolates have shown bridging reactions, for instance, with AG-8 and AG-BI. Furthermore, a hyphal fusion event in which the cell walls of hyphae fuse without membrane contact falls into an incomplete category (Sneh et al., 1996). However, it is possible that AGs alone will not reveal the genetic diversity or taxonomic connections among *Rhizoctonia* isolates under these conditions. It’s also worth noting that, except AG-2-1 and AG-2-2, isolates belonging to various subgroups within a single AG may anastomose with one another; thus, this is not a reliable method for distinguishing across AG subgroups.

### 5.2. Biochemical approaches

Fatty acid profiling, pectin enzyme analysis, allozyme polymorphism, and serological methods are the four main approaches that have been developed so far (Banniza and Rutherford, 2001). Fatty acid profiling *R. solani* AGs were characterized by gas chromatography and analyzed with Microbial Identification System software. Several fatty acids, such as linoleic, palmitic, stearic, and oleic, quantities and presence or absence, could be used to distinguish *R. solani* AG. However, fatty acids of subgroups, for example, AG-1-IA and AG-1-IB, were very similar. Hence fatty acid profiling cannot distinguish AG subgroups of *R. solani* (Johnk and Jones, 2001). Therefore, there were chances of misidentification of AG subgroups.

Pectic enzyme analysis was also used to diagnose *R. solani*. Polygalacturonases (PG) are glycosyl hydrolases that catalyze the random hydrolysis of 1, 4 -D-galacturosiduronic links in pectate and other galacturonans. Many phytopathogenic fungi use PG as a virulence factor. This enzyme has been used as an *R. solani* grouping and detection marker. Pectic zymograms were utilized to characterize AGs associated with legumes. For example, *R. solani* AG-4 isolates infecting common beans have also been described and grouped using pectic enzyme analysis (O’Briert and Zamani, 2003).

In AGs and subgroups of *R. solani*, genetic diversity studies using isozyme analysis have been used as a discriminative technique, as found in a study reported by (Mahmoud et al., 2007). Using different enzyme systems, they found three groups in AG-2 isolates of *R. solani*. Cluster analysis and data were used from seven enzyme systems to divide 23 *R. solani* AG-1 isolates into three groups. Similarly, isozyme analysis was utilized (Laroche et al., 1992) to differentiate AG-3 and AG-9 in *R. solani*. Furthermore, isozyme analysis was used to distinguish AG-11 isolates from Australia and Arkansas (Kaufman and Rothrock, 1995).

*Rhizoctonia solani* can be detected using antibodies that bind to fungal mycelium in a species-specific manner (O’Briert and Zamani, 2003). An ELISA reaction, in which the antibody is introduced to the wells of a microtiter tray coated with antigen, is the most systematic way to detect binding. The addition of a second enzyme-linked anti-immunoglobulin antibody that binds to the antigen–antibody complex detects specific binding of the antibody to the antigen. The quantity of enzyme activity trapped in the wells is proportional to the antigen concentration. Antibody detection tests for *R. solani* AG-3 have been produced (Thornton et al., 1993). The lack of specificity of the antibody may be a drawback of antibody tests. Antibodies are not always specific enough to distinguish pathotypes of the same species or even closely related ones (Paulitz and Schroeder, 2005).

Besides, these methods, commonly not used for direct soil or plant material testing, require skilled persons for proper detection. They appear to be particularly useful for examining genetic diversity among AGs but cannot differentiate AG subgroups of *R. solani* (Budge et al., 2009). A fast, precise, and cost-effective approach for identifying pathogens like *R. solani* is matrix-assisted laser desorption ionization-time of flight mass spectrometry (MALDI-TOF MS; Chun et al., 2022). MALDI-TOF MS is a desorption technique for “soft” ionization, in which the analyte is ionized in minute fragments. Matrix molecules are crystalline and work to shield the analyte from the harmful effects of the laser light while yet allowing it to ionize it. The synaptic, −cyano-4-hydroxycinnamic, and 2,5-dihydroxybenzoic acids are utilized as a matrix. Non-volatile, high-molecular-weight molecules are a common target for MALDI-TOF mass spectrometry’s study (peptides, proteins, carbohydrates, oligonucleotides, synthetic polymers, complex organic compounds, etc.). In a study, 20 proteins associated with sclerotia of *R. solani* were found to be differentially expressed in the first round of 1-DE using MALDI-TOF MS, and another 55 proteins were found in 2-DE using either MALDI-TOF MS or MALDI-TOF/TOF MS (Kwon et al., 2014). Genetic information processing, carbohydrate metabolism, cell defense, amino acid metabolism, nucleotide metabolism, cellular processes, pathogenicity and mycotoxin production, and hypothetical or unknown functions are among the 10 categories into which the identified proteins have been placed based on their biological functions. The results of this study add to our knowledge of the biology of *R. solani* sclerotia.

### 5.3. Molecular approaches

Numerous pathogen detection techniques based on nucleic acid markers are generally divided into the following categories: (a) Genomic complementarity by DNA–DNA hybridization assays; (b) PCR analyses with random primers, such as Random Amplified Polymorphic DNA (RAPD; Duncan et al., 1993), Amplified Fragment Length Polymorphism (AFLP), etc.; and (c) PCR analyses with sequence-specific primers, such as ITS and Intergenic Spacer (IGS It has been reviewed how some of those methods are used to classify and phylogeny plant pathogenic oomycetes and true fungi. Results obtained by putting *Rhizoctonia* species in phylogenetically distinct clades supported the idea of AGs for *Rhizoctonia* species. Using these techniques, some *R. solani* AGs were found to have genetically distinct subgroups (Chavarro-Mesa et al., 2020; Misawa et al., 2020).

#### 5.3.1. Polymerase chain reaction-based approaches

Due to high sensitivity, polymerase chain reaction (PCR)-based technologies have widespread applications (Bounou et al., 1999). The low copy number of DNA may be amplified millions of times with excellent sensitivity using PCR, which aids in diagnosis. DNA sequence-specific PCR primers have been developed that may pick up on low concentrations of *R. solani* DNA in plant and soil material (Liu et al., 1995). *R. solani* AGs such as AG-1-IB (Grosch et al., 2007), AG-2 and subgroups (Salazar et al., 2000), AG-3 in potatoes (Bounou et al., 1999), AG-4 and AG-8 in wheat (Brisbane et al., 1995), and AG-8 in soil have all been detected using PCR techniques (Whisson and Francis, 1995). However, a species-specific diagnostic kit for *R. solani* is challenging to create because of the heterogeneous nature of its AGs.

The detection technique developed by Bounou et al. (1999), which is based on PCR and uses just one restriction endonuclease (Xho I), is specific, reliable, and applicable to both plant tissue and soil (Pannecoucque and Hofte, 2009). Similar results were reported by Pannecoucque and Hofte (2009), who employed the enzymes Ava II and/or Hinc II for PCR RFLP analysis and discovered that *R. solani* isolates that are genetically distinct while belonging to the same cluster in a sequence-based classification. For this purpose, rRNA-encoding genes are analyzed. Taxonomic and phylogenetic studies using conserved genes with enough sequence variation are often used in developing PCR diagnostic tests. Ribosomal RNA gene sequences have been employed extensively in phylogenetic analyses of fungi (Cubeta et al., 1996). Depending on the kind of fungus, these genes are often located in the mitochondria or the nucleus (Gardes and Bruns, 1993). Hundreds of tandem repeats surround each fungal nuclear rRNA gene unit, which is bordered by three other genes: the tiny rRNA genes 18S and 5.8S and the great rRNA gene 28S (Capote et al., 2012). The use of these genes in fungal taxonomy and phylogeny is common (Vilgalys and Gonzalez, 1990). The sequences of the large and small subunits are conserved, but the portions of the internal transcribed spacer (ITS) that separate them are variable and may be utilized to tell closely related species apart (Gardes and Bruns, 1993).

*R. solani* AG-4, as reported by Vilgalys and Gonzalez (1990), has rDNA repeats of about 8.8 kb and a consistent rDNA copy number of 59 per haploid genome. The ITS region (ITS1-5.8S-ITS2) sequencing database of *R. solani* is widely accessible in gene banks, and this ITS database aids in *R. solani* phylogenetic study. The 18S and 28S subunits sequencing of *R. solani* isolates are also publicly accessible. Genetic links within an AG and occasionally within AG subgroups were studied using the rRNA gene sequences, confirming their genetic uniqueness. DNA/DNA hybridization, RFLP analysis, PCR fingerprinting, and other techniques were also used to validate their genetic identity (Capote et al., 2012). Analysis of DNA sequence complementarity by observing the degree of DNA hybridization between DNA molecules of various isolates was one of the earliest molecular techniques used to establish the genetic relatedness of *R. solani* isolates (Kuninaga and Yokosawa, 1985). The DNA–DNA hybridization values obtained for isolates of the majority of *R. solani* AGs, including AG-2-1, AG-3, AG-5, AG-7, AG-8, and AG-BI, were found to be at a very high level (90%). However, some AG groups’ subgroups exhibit significant variations in DNA hybridization values, such as the subgroups of AG-1, AG-2-2, and AG-9. These subgroups within an AG have relatively low genetic relatedness (47–87%), which fits in well with previously established subgroups based on morphology, pathogenicity, and vitamin requirement. Additionally, DNA–DNA hybridization assisted in the discovery of previously undiscovered AG-4 and AG-6 subgroups. Isolates of the HG-I and HG-II subgroups of AG-4 hybridized at low levels of 30 to 47%. The relatedness of isolates from subgroups HG1 and GV within AG-6 ranged from 47 to 62%.

The 5.8S region is entirely consistent across all datasets; however, the internal transcribed spacers (ITS) show a lot of diversity (Carling et al., 2002). When comparing the ITS region to the large subunit area, Gonzalez et al. (2001) discovered that the ITS region was more variable and more difficult to align. Changes in biological properties like pathogenicity and habitat are correlated with variations in ITS regions and rDNA nucleotide sequence. All ITS sequences had the same components—ITS1, ITS2, and 5.8S rDNA—and the same 3′ and 5′ ends of 18S and 28S rDNA. It is possible to amplify ITS DNA from *R. solani* by PCR using purified fungal DNA using ITS1 and ITS4 primers (Jaaffar et al., 2016; Misawa et al., 2018).

#### 5.3.2. Real-time PCR

Real-time PCR is now commonly acknowledged as one of the most sophisticated identification methods for plant pathogen detection. It can detect and amplify small quantities of DNA in the sample. Quantitative polymerase chain reaction (qPCR) also has been reported to use for taxon-specific quantification (Bass et al., 2015). In addition to providing quantitative data, qPCR has greater sensitivity and a fast-sampling rate than traditional PCR. SYBR Green, unique fluorescent-labeled probes like TaqMan, Molecular Beacons, or Scorpions, or dye-primer dependent systems such as hairpin primers or the Plexor system may all be opted in RT-PCR chemistry (Oliveira et al., 2018). The complexity of RT-PCR and issues with sensitivity, repeatability, and specificity are disadvantageous. Furthermore, when employed as a quantitative approach, it suffers from the same issues as classical PCR (Bustin, 2000).

#### 5.3.3. SCAR approach

A PCR-based tool called ‘sequence characterized amplified region’ (SCAR) is designed to amplify members from the same genus. It is also reported that these markers work more efficiently at the genus level than at the species level. When intra-species evolutionary relationships are strong, this method can diagnose members at the genus level with high specificity using soil or infected plant parts. Moreover, this tool is used to detect disease-causing agents quickly to manage the disease. Identifying these SCAR markers at an AGs level is suggested for better results (Lübeck and Lübeck, 2005).

#### 5.3.4. PCR fingerprinting techniques

The PCR fingerprinting methodology can amplify random tandem repeats on genomic DNA. It can detect species-specific patterns (McCartney et al., 2003). Fingerprinting methods also find the phylogenetic structure of different microbial species. To estimate genetic variations among AGs of *R. solani*, PCR finger-printing protocols such as (randomly amplified polymorphic DNA (RAPD), DAF (DNA Amplification fingerprinting techniques; Patil and Solanki, 2016), amplified fragment length polymorphism (AFLP; Julián et al., 1999), restriction fragment length polymorphism (RFLP; Vilgalys and Gonzalez, 1990) and universally primed PCR (UP-PCR) are also used (Lübeck and Poulsen, 2001). These methods permit accurate and rapid AGs and AG sub-group identification leading to an improved understanding of population structure. Universally primed PCR (UP-PCR) allows rapid identification of AG subgroups in a cross-hybridization assay.

Besides, these methods do not require prior knowledge about primer sequences; therefore, it is advantageous for poorly investigated genomes. The methods require short (5–20 nucleotides), arbitrary oligonucleotide primers to obtain discrete sequence unknown DNA segments of variable lengths (Lakshman et al., 2016). The PCR fingerprinting techniques utilize a single primer of 10 nucleotides to obtain multiple copies of DNA fragments of varying sizes. Additionally, the UP-PCR technique has been used to assess genetic differences of AG subgroups of *R. solani* (Bulat et al., 1998). The AFLP approach is slightly different from the above approaches and is more effective in detecting inter and intra-specific genetic variation of *R. solani* than RFLP. The number of bands produced by AFLP varied between 50 and 70. This method has two advantages: excellent repeatability and a higher proportion of every reaction’s genome analyzed. However, PCR fingerprinting approaches necessitate pure fungal cultures and are not ideal for directly exploring plant material, soil, or growing media. Hence, the invention of PCR primers that recognize unique DNA sequences can now detect meagre quantities of target DNA in plant material and soil (Liu et al., 1995).

#### 5.3.5. Cross-hybridization

In one application of universally primed polymerase chain reaction (UP-PCR), called cross-hybridization, a single UP-primer is used to determine the sequence similarity (homology) of unknown *Rhizoctonia* strains in comparison to UP-PCR hybridization groups (Lübeck and Lübeck, 2005). First, UP-PCR products from many strains are blotted onto a membrane; secondly, UP-PCR products from a reference strain are used as a hybridization probe since they can be readily labelled. This technology’s main benefit is its ability to simultaneously evaluate several strains’ sequence homology. Probe DNA may be marked with either radioactive phosphorous or a nonradioactive compound like digoxigenin (DIG). The intensity of the hybridization signal is used to establish the strain’s relationship to the mystery strain. After an hour, signals from the radioactive probe are evident on the autoradiograph, proving that the hybridized strains are all part of the same UP-PCR hybridization group. A low signal strength indicates a low degree of similarity, and a nonexistent signal strength indicates no relationship between the tested strains. Nonradioactive detection is identical to antigen–antibody interactions (Lübeck and Poulsen, 2001). Before the advent of the UP-PCR cross-hybridization assay, a single UP primer was needed to discover and classify 21 *Rhizoctonia* isolates into 11 AGs swiftly. Among isolates from the same AG subgroup, they found extensive cross-hybridization, but amongst different AGs, they found very little or none at all. In addition, total DNA–DNA hybridization and this approach were employed to identify 16 *Rhizoctonia* isolates that were determined to be comparable (Lübeck, 2004).

#### 5.3.6. Transcriptomic approaches

New information on closely related strains of *R. solani* with distinct characteristics may be gleaned through comparative sequencing analysis. The AG-1 IA strain, lettuce’s AG-1 IB, AG-7, AG-3, AG-14, sugar beet AG-2-2 IIIB, potato AG-3, and lupin AG-8 have all generated draft genome sequences for them (Wibberg et al., 2016). The intraspecific genomic variability of *R. solani* makes it challenging to compare genetic data, and the fragmented nature of these genome sequences does not help matters. So, *de novo* transcriptome sequencing is helpful for studying *R. solani*’s genetic makeup and variety (Zheng et al., 2013). Construction of a basal transcriptome profile (without inoculation on host plants) of the AG-1-IA strain might provide unique insights into the genes implicated in the pathogenesis of rice sheath blight since host immunity impacts the control of gene expression in *R. solani*.

In addition, the AG-1-IB and AG-1-IC mycelial transcriptome sequences are valuable tools for studying the commonalities and differences in genetic, physiological, and pathogenic features within the AG-1 subgroup. To further understand this phytopathogen complex, researchers performed an integrated transcriptome analysis for the AG-1-IA, AG-1-IB, and AG-1-IC subgroups (Xia et al., 2017). Differential sequencing indicated deep historical chasms within the AG-1 gene family. Similar traits were seen in AG-1 simple sequence repeats in transcripts, although polymorphic sites were also present. AG-1 showed intra-subgroup polymorphisms that were consistent with different levels of genic heterozygosity. There was similarity in the sequences of putative pathogenic factors, enzymes involved in the production of phytotoxins, secreted lignocellulosic enzymes, enzymes involved in the detoxification of reactive oxygen species, and apoplastic/cytoplasmic effector possibilities (Mat Razali et al., 2021). Differentiation of AG-1 subgroups has led to the emergence of Cys-rich tiny secreted proteins, as shown by a secretome subset’s dN/dS ratio. By identifying allergy protein homologs, oxidative phosphorylation and ethylene biosynthesis pathways, and diversification of polysaccharide monooxygenases, we gain molecular insight into critical genomic components that play a role in *R. solani* pathogenesis (Yamamoto et al., 2019). Genetic investigations of *R. solani* need intensive work with massive sequencing data because of the species’ multinucleate nature.

#### 5.3.7. Genomic approaches

*R. solani* AG-1-IA, found in infected rice in South China, has a draft genome sequence of 36.94 Mbp. This sequence was assembled into 2,648 scaffolds (with an N50 scaffold size of 474.5 Kb), and 6,156 genes were annotated (Zheng et al., 2013). This work aimed to sequence four *R. solani* genomes isolated from rice infected with *R. solani* and perform comparative genomic studies among them (*R. solani* AG-1-IA and four *R. solani* genomes belonging to AGs other than AG-1-IA; AG-1-IB, AG-2, AG-3, and AG-8). In addition, analyses revealed that the genomic conservation of *R. solani* genomes among neighboring AGs was more diverse than among AG-1-IA isolates and that the presence of numerous predicted pectin modification genes in the rice-infecting *R. solani* genomes may contribute to the extensive host range and virulence of this necrotrophic fungal pathogen (Lee et al., 2021).

#### 5.3.8. Loop-mediated isothermal amplification

The lack of spores, often used for identifying and classifying fungi, makes it challenging to identify *R. solani* accurately. Diagnosis times may be cut drastically by using techniques that can be carried out in the field. Loop-mediated isothermal amplification (LAMP) is one such technique, and it may be done in under an hour for very little money by employing a water bath or heating block (Tomlinson et al., 2010). Although PCR generates vast quantities of DNA by temperature cycling, LAMP uses a constant temperature to produce DNA. The DNA polymerase used in LAMP is a big fragment with 5′3’ polymerase activity but no 5′3’ exonuclease activity. Two pairs of primers, called the inner (FIP, Forward Inner Primer, and BIP, Backward Inner Primer) and outer (F3 and B3), are often used in LAMP methods. The LAMP process employs all four primers initially; however, only the inner primers are necessary for strand displacement DNA synthesis (Notomi et al., 2000). Results from positive LAMP responses are evaluated visually by seeing the buildup of a yellowish residue. Quantitative readouts, however, are necessary to enhance the interpretation and field usability of LAMP approaches since visual evaluation might be equivocal.

To detect *R. solani*, a highly sensitive and easy-To-Use LFD-based LAMP assay were developed (Patel et al., 2015). Both *R. solani* and *R. zeae* were found using this approach In many plants and soil samples. In this investigation, The researchers determined that even trace amounts of *R. solani* and *R. zeae* DNA could Be identified using The LAMP assay. Compared To The estimated 87.1 mb diploid genome size of *R. solani*, The number of copies of The genome that Can Be identified using The LAMP procedure proposed In this work Is very small. This offers a compassionate approach To detection that may Be used On tissues with subclinical *R. solani* infection that have Yet To show Any clinical symptoms (Wibberg et al., 2016).

#### 5.3.9. Next-generation sequencing technologies

Especially in the presence of members of closely related families such as AGs, morphological traits alone may not be sufficient for reliably recognizing pathogens similar to *R. solani*. It has been shown that comparing sequencing data provided by PCR amplification of a target gene using universal primers amplifying a conserved portion with reference databases is an efficient strategy for finding new fungi. However, there are possible limitations to employing DNA similarity-based sequence databases, such as insufficient sequences, sequences linked with misidentified species, the difficulty to edit or update data readily, and problems with defining species boundaries, all of which may lead to a misunderstanding of search results (Kang et al., 2014).

The Sanger sequencing technique, which can produce several short sequences from a variety of species in a short period, has superseded or updated several “next-generation” sequencing methods. Because of the tremendous advances in commercial sequence throughput made possible by massive sequencing technology, genomic research has been profoundly impacted. For instance, the lupin pathogen *R. solani* AG-8 isolate WAC10335 has a high-quality genome validated by RNA-seq and a thoroughly curated list of 13,964 genes (Hane et al., 2014). AG-8 had a higher incidence of heterozygous SNP mutation within a single isolate than was reported in fungal populations. By comparing AG-8 to 308 proteins with effector-like properties, researchers could make educated guesses about the biological processes involving these proteins. Predicted effector-like proteins have a larger ratio of non-synonymous to synonymous point mutations (dN/dS) than other proteins, suggesting that they are subject to several selective pressures. The public has also gained access to extensive genomic materials for *R. solani* AGs beyond the expressed sequence tags (ESTs) libraries of AG-1-IA and AG-4 (Lakshman et al., 2016).

#### 5.3.10. Nanopore sequencing

When compared to other sequencing technologies, nanopore sequencing is more intuitive. In this experiment, we introduce a single, tiny hole into an insulating membrane and apply an electrical potential. The sequence is determined by drawing a DNA strand through the hole and seeing how the various base combinations affect the current flow. David Deamer first conceived this notion as we knew it now in 1989, but it required more than two decades of significant advancements to bring it to life (de Lannoy et al., 2017). Since ONT’s MinION was the first commercially available nanopore sequencing device in 2014, and the MinION Access Program (MAP) began that same year, there has been rapid progress in the field of nanopore sequencing, with new applications and improvements to existing ones being published regularly (Jain et al., 2018). The MinION has several benefits over competing sequencing machines. Its $1,000 starting price is a fraction of that of rivals, and its compact size (about the size of a smartphone) makes it easy to transport and store. The cost of running the MinION is low and manageable; for example, a 48-h sequencing run may cost as little as $800 and produce as much as 5 Giga bases of raw sequenced data (Liu et al., 2021b). Further, unlike Sanger sequencing, second-generation sequencing, and the SMRT method, which all need labelling of nucleotides to distinguish between them, this approach does not depend on labelling techniques to identify distinct bases. Unlike the Sanger and SGS procedures, the MinION allows you to skip the process of amplification by PCR. Simplifying sample preparation for MinION samples by skipping these stages helps to reduce mistakes and biases (e.g., the CG bias for PCR). Thus, it may be used to identify bases that have been altered (Simpson et al., 2016). Last but not least, the maximal read length generated by the MinION is far longer than that of next-generation and Sanger sequencing and is only matched by SMRT sequencing. This is particularly useful for resolving repetitive sequences. The MinION’s poor signal-to-noise ratio, the randomness imposed by its biological components, and the ensuing high mistake rate of base calling are its main drawbacks compared to its rivals (de Lannoy et al., 2017). A complex fungal genome was reconstructed from nanopore readings alone (Datema et al., 2016). They were able to generate a highly contiguous draft genome sequence from a modest sequencing depth after isolating ultra-pure high molecular weight (HMW) DNA from *R. solani*. They have decreased the number of contigs by order of magnitude and raised the N50 contig size to slightly around 200 kb, compared to earlier assembly. Their findings suggested that almost complete eukaryotic genomes of excellent quality are feasible with minimal resources.

## 6. Management approaches

The fungal pathogen *R. solani* causes several diseases in legumes and other hosts. Many techniques are used to control the pathogenicity of this fungal species, including some conventional and biotechnological approaches, as described below.

### 6.1. Conventional approaches

In the past, general or non-specific approaches, including planting tricks, soil fumigation, and soil moisture, were used to control *Rhizoctonia* infection. Methyl bromide and metam sodium were used for soil fumigation (Duniway, 2002). Seed coating was done with pesticides and fungicides using captafol, thiram, pencycuron, etc. However, these compounds were not successfully used in growing years because they were costly and harmful to the environment. Moreover, agricultural practices planting tricks, and irrigation intervals were more effective for controlling *R. solani* than the previously mentioned approaches, either alone or in combination (Narayanasamy, 2011).

### 6.2. Biological approaches

The identification and molecular characterization of microorganisms to control fungal diseases are highly relevant in eco-compatible agriculture (Spadaro and Gullino, 2014). Antibiosis, direct parasitism, competition, hypovirulence, suppression, induced resistance, and predation are some mechanisms that can be used to control plant diseases biologically. Antibiosis is an antagonistic reaction caused by metabolites of fungal antibiotics or antibiotic-like compounds such as lytic enzymes, volatile compounds, siderophores, or other toxic substances (Kuramae et al., 2007).

Different plant cell wall degrading enzymes, such as cellulases, proteases, chitinases, and α-1,3-glucanases, have been shown to have antifungal activity in the literature (Ozbay and Newman, 2004). With the help of some bacterial biocontrol agents, siderophores play an essential role in suppressing plant diseases. These compounds inhibit pathogens’ growth and metabolic activity by sequestering iron (Haggag et al., 2007). Moreover, volatile ammonia is also reported as an inhibitory mechanism to control diseases caused by *R. solani* and other pathogens (Paulitz et al., 2000).

Some promising “Biological Control Agents” (BCAs) are important to control diseases caused by *R. solani*, most notably *Bacillus* spp., *Rhizobium*, *Serratia*, *Streptomyces*, *Pseudomonas*, *Trichoderma,* and other genera, have been demonstrated in recent research (Yobo et al., 2010). According to Montesinos (2003), patented biopesticides (biocontrol agents) are typically made of bacteria, followed by fungi. BCAs have many advantages, including long-term disease suppression, self-sustainability, and the ability to spread once established (Montesinos, 2003).

Besides organic amendments, e.g., animal manure, compost, and organic industrial products have been well addressed to suppress fungal pathogens (Noble and Coventry, 2005). Damping-off of flax is reduced by mushroom compost and manure, with the compost being more effective than the manure (Alabouvette et al., 2004). Compost residues obtained from composted cow manure and viticulture and enological factories significantly reduced the damping-off of cress (*Lipidium sativum*) caused by *R. solani* (Pane et al., 2011). Besides, soil amendments obtained from agricultural waste composts also reduce disease risk caused by several soil-borne plant pathogens such as *Rhizoctonia* spp. (Rivera et al., 2004), *Pythium* spp. (Pascual et al., 2000), *Fusarium* spp. (Cotxarrera et al., 2002), and *Phytophthora* spp. (Widmer et al., 1999). Nanoforms of silver, copper, silica silver, and carbon are some of the nanoparticles used in crop protection. According to (Park et al., 2006), crop protection using nanotechnology methods, such as silica-silver 10 ppm, resulted in the most effective inhibition of *R. solani* growth. The growth and development of gram-positive and gram-negative bacteria can be suppressed by Nano-sized silica silver. Moreover, nano-copper is very effective for controlling bacterial diseases like mung bean leaf spot (*X. campestris* pv. *phaseoli*) and rice bacterial blight (*Xanthomonas oryzae* pv. *oryzae*). However, compared to biological, nanoparticle synthesis has physical and chemical limitations in maintaining shape, size, and monodispersity. *Trichoderma* (teleomorph: *Hypocrea*) is a soil fungus. Numerous *Trichoderma* spp. are used as biocontrol agents (BCAs) to control fungal pathogens. A researcher examined *Trichoderma* treatment to control *R. solani* by producing ergosterol and squalene in bean plants (Mayo et al., 2015). Their production levels differed among the *Trichoderma* isolates; T019 was found with a higher level of both compounds. Moreover, T019 increases the resistance level of bean plants against *R. solani* and promotes the expression of plant defense related genes (Infantino et al., 2006).

### 6.3. Resistant sources of legumes against *R. solani*

*R. solani* affects various leguminous crops around the world. Different methods, *viz.* biological, chemical, and cultural practices, are adopted to control fungus from the field. Some resistant sources in legumes are also reported against *R. solani,* for example, in lentils (Infantino et al., 2006).

Integrated control of *R. solani* in leguminous crops through resistant genotypes cultivation is a good strategy. Still, some resistant sources have been reported in faba beans to cope with this pathogenic fungus (Infantino et al., 2006). Moreover, 304 faba bean genotypes in Canada were grown, but only five were resistant to *R. solani* (Assunção et al., 2011). It is suggested to increase the durability of resistance against *R. solani* using complementary management strategies. These strategies include crop rotation, incorporating organic matter, and letting the sunshine on the soil before planting.

The inability of *R. solani* to form infection cushions and penetrate the hypocotyl has been reported to make mature red kidney bean plants resistant to *R. solani* -caused diseases (Keshavarz Tohid and Taheri, 2015). Increased calcification of cell walls and increased cuticle thickness are two critical factors in this resistance. Furthermore, infection cushion formation requires exudates reduced by the thicker cuticles of older plants. Therefore, older bean plants can be resistant because the quantities of the exudates are insufficient to stimulate the formation of infection cushions (Contina, 2016).

Kelly et al. (2003) reported that the common bean has many resistance genes against the diseases caused by *R. solani* (Kelly et al., 2003). These genes are found in groups at complex loci. The physical arrangement and sequence diversity of fungal disease-resistant gene families must be understood. Cloning of resistance gene analogs (RGAs) and the development of targeted region amplified polymorphisms (TRAP) have become essential approaches for analyzing resistant genes (Hu and Vick, 2003).

Another group of *Rhizoctonia* species is binucleate *Rhizoctonia* (BNR), classified into 16 AGs (Sharon et al., 2008). Several BNR species AGs have been reported that affect essential crops worldwide (Kuramae et al., 2007). However, numerous studies have demonstrated the ability of BNRs to protect leguminous crop seedlings from various causal agents, *R. solani* and *Pythium* spp. (Wen et al., 2005). Moreover, BNRs can prevent diseases of arabidopsis and soybean caused by *R. solani* (Sharon et al., 2011).

## 7. Conclusion and future perspectives

*R. solani* is an important plant pathogen that causes yield losses in legume crops worldwide. *R. solani* affects plants of different families, such as *Araceae, Amaranthaceae, Linaceae*, *Moraceae*, *Malvaceae*, *Fabaceae*, *Poaceae*, *Rubiaceae,* and *Solanaceae.* Among these families, legume crops in the *Fabaceae* family are severely infected by *R. solani*. *R. solani* has distributed in all legume-growing regions of the world. This review outlines traditional, biochemical, molecular, genomic, transcriptomic, and NGS-based detection and diagnostic approaches of *R. solani* associated with the most economically important legume crops. Traditional approaches are based on incubation, grow-out procedures, morphology and microscopy identifications, and visual examination of symptoms in legume crops. Although they are often employed due to their convenience of application, they are time-consuming, require mycological expertise, are sometimes not sensitive enough to low levels of inoculum, and are not always reliable. However, molecular approaches allow for more precise, practical, and reproducible identification of pathogens at the genus, species, AGs, and AG subgroup levels. Molecular approaches have trouble producing a high-quality DNA template because of PCR inhibitors and cannot distinguish between viable and non-viable inoculum of *R. solani*. This problem has been avoided and improved the detection of *R. solani* by developing novel PCRs. Recently, improvements in “omics” research will also open up new avenues for studying legumes-*R. solani* interactions, establishing disease epidemiology and creating cutting-edge methods for detecting and diagnosing *R. solani*. Genomic and comparative genomic (e.g., transcriptomic, proteomic, and metabolomic) studies on *R. solani* currently being conducted in laboratories around the world should be able to identify genes for pathogenicity factors and candidate targets for interfering with the disease process and on critical steps of the pathogen’s survival. Insights into the phylogenetic diversity and relatedness among the *R. solani*, AGs, and subgroups can be gained through comparative genomics. Recently whole genome sequencing (WGS) of *R. solani* AGs has assisted us in pinpointing the most responsible genes for observed differences in host range, pathogenicity, overwintering, competitive saprophytic, aggression, and epidemiological fitness. Further insight into mitochondrial inheritance and genome plasticity should be gained from comparative mt-genome analysis. Moreover, new probes and markers have been identified for studying genetic variation in *R. solani* populations. Genome sequence data will help researchers learn about sensitivity to fungicides, prevent the development of fungicidal resistance, and create new fungicides with less impact on the environment. The mating habits, gene flow, and geographical distribution of *R. solani* genetic variants can all be better understood with the help of data gathered from population genetics studies. Unique and effective detection and diagnostic approaches such as NGS and WGS are increasingly emerging. So far, these approaches are not highly used for *R. solani* diagnostics, but they will likely become more utilized in detecting and diagnosing *R. solani*. With this newfound knowledge, we can hopefully better detect, diagnose, and develop novel sustainable strategies to control *R. solani*.

## Author contributions

MAA, SWK and RK: writing initial draft. AA and MM: writing final draft, software, and figure preparations. MAS, MKS and PKD: collecting literature, tables preparations, and editing. LZ: supervision, validation, and finalized the review. All authors have read and agreed to the published version of the manuscript. All authors listed have made a substantial, direct, and intellectual contribution to the work and approved it for publication.

## Funding

This work was supported by the High-talent Introduction and Continuous Training Fund to LZ (grant no: 10300000021LL05) and Discipline Construction Funds (grant no: 10407000019CC2213G), supported by Zhejiang Academy of Agricultural Sciences (ZAAS) and State Key Laboratory for Managing Biotic and Chemical Threats to the Quality and Safety of Agro-products (10417000022CE0601G/029).

## Conflict of interest

PKD was employed by Contec Global Agro Limited.

The remaining authors declare that the research was conducted in the absence of any commercial or financial relationships that could be construed as a potential conflict of interest.

## Publisher’s note

All claims expressed in this article are solely those of the authors and do not necessarily represent those of their affiliated organizations, or those of the publisher, the editors and the reviewers. Any product that may be evaluated in this article, or claim that may be made by its manufacturer, is not guaranteed or endorsed by the publisher.

## References

[ref1] Abbas A. Mubeen M. Sohail M. A. Solanki M. K. Hussain B. Nosheen S. (2022a). Root rot a silent alfalfa killer in China: distribution, fungal, and oomycete pathogens, impact of climatic factors and its management. Front. Microbiol. 13:961794. doi: 10.3389/fmicb.2022.961794, PMID: 36033855 PMC9403511

[ref2] Abbas A. Mubeen M. Zheng H. Sohail M. A. Shakeel Q. Solanki M. K. (2022b). *Trichoderma* spp. genes involved in the biocontrol activity against *Rhizoctonia solani*. Front. Microbiol. 13:884469. doi: 10.3389/fmicb.2022.884469, PMID: 35694310 PMC9174946

[ref3] Abd El-Naby Zeinab M. Azzam C. R. Abd El-Rahman S. S. (2014). Evaluation of ten alfalfa populations for forage yield, protein content, susceptibility to seedling damping-off disease and associated biochemical markers with levels of resistance. J. Am. Sci. 10, 73–85. doi: 10.7537/marsjas100714.11

[ref4] Abd-Elmagid W. M. Aly M. M. E.-S. El-Sharkawy R. M. (2020). Control of peanut root and pod rots diseases using certain bioagents. J. Phytopathol. Pest Manag. 7, 79–90.

[ref5] Abdel-Monaim M. F. (2011). Integrated management of damping-off, root and/or stem rot diseases of chickpea and efficacy of the suggested formula. Not. Sci. Biol. 3, 80–88. doi: 10.15835/nsb336134, PMID: 36040226

[ref6] Ahmad R. Z. Ameen F. Khalid R. Alghuthaymi M. A. Alsalmi R. Li C. (2019). A brief history of endophyte detection techniques in grasses. Sustain. Agric. Res. 8, 66–72. doi: 10.5539/sar.v8n3p66

[ref7] Ahmad R. Khalid R. Aqeel M. Ameen F. Li C. (2020). Fungal endophytes trigger *Achnatherum inebrians* germination ability against environmental stresses. S. Afr. J. Bot. 134, 230–236. doi: 10.1016/j.sajb.2020.01.004

[ref8] Ajayi-Oyetunde O. O. Bradley C. A. (2017). Identification and characterization of *Rhizoctonia* species associated with soybean seedling disease. Plant Dis. 101, 520–533. doi: 10.1094/PDIS-06-16-0810-RE, PMID: 30677363

[ref9] Ajayi-Oyetunde O. O. Bradley C. A. (2018). *Rhizoctonia solani*: taxonomy, population biology and management of *rhizoctonia* seedling disease of soybean. Plant Pathol. 67, 3–17. doi: 10.1111/ppa.12733

[ref10] Alabouvette C. Backhouse D. Steinberg C. Donovan N. Edel-Hermann V. Burgess L. (2004). “Microbial diversity in soil-effects on crop health,” in Book Managing Soil Quality: Challenges in modern agriculture. eds. Schjønning P. Elmholt S. Christensen B. T. (Wallingford, UK: CABI Publishing), 121–138.

[ref11] Al-Askar A. Ghoneem K. Rashad Y. (2013). Management of some seed-borne pathogens attacking alfalfa plants in Saudi Arabia. Afr. J. Microbiol. Res. 7, 1197–1206. doi: 10.5897/AJMR12.739

[ref12] Al-Askar A. A. Rashad Y. M. (2010). Efficacy of some plant extracts against *Rhizoctonia solani* on pea. J. Plant Prot. Res. 50, 239–243. doi: 10.2478/v10045-010-0042-0, PMID: 35237287

[ref13] Alsohim A. S. (2020). Influence of *Pseudomonas fluorescens* mutants produced by transposon mutagenesis on in vitro and in vivo biocontrol and plant growth promotion. Egypt. J. Biol. Pest Control. 30, 1–9. doi: 10.1186/s41938-020-00220-5

[ref14] Amani A. Fatma M. El-Demerdash (2017). The ameliorative effects of silicon on salt-stressed sorghum seedlings and its influence on the activities of sucrose synthase and PEP carboxylase. J. Plant Physiol. Pathol. 5, 1–8. doi: 10.4172/2329-955X.1000164

[ref15] Andersen A. Huber D. (1965). Plate-profile technique for isolating soil fungi and studying their activity in vicinity of roots. Phytopathology 55:592.

[ref16] Assunção I. P. Nascimento L. D. Ferreira M. F. Oliveira F. J. Michereff S. J. Lima G. S. (2011). Reaction of faba bean genotypes to *Rhizoctonia solani* and resistance stability. Hortic. Bras. 29, 492–497. doi: 10.1590/S0102-05362011000400008

[ref17] Bai Q. R. Jiang X. Y. Xie Y. Y. Sun H. Y. Gao J. (2014). Summer blight of white clover (*Trifolium repens*) caused by *Rhizoctonia solani* AG-1-IB in China. Plant Dis. 98, 1153–1153. doi: 10.1094/PDIS-09-13-0987-PDN, PMID: 30708801

[ref18] Banniza S. Rutherford M. A. (2001). Diversity of isolates of *Rhizoctonia solani* AG-1 1A and their relationship to other anastomosis groups based on pectic zymograms and molecular analysis. Mycol. Res. 105, 33–40. doi: 10.1017/S0953756200003348

[ref19] Basbagci G. Dolar F. S. (2020). First report of binucleate *Rhizoctonia* AG-K causing root rot on chickpea. Arch. Phytopathol. Plant Protect. 53, 640–652. doi: 10.1080/03235408.2020.1789822

[ref20] Bass D. Stentiford G. D. Littlewood D. T. J. Hartikainen H. (2015). Diverse applications of environmental DNA methods in parasitology. Trends Parasitol. 31, 499–513. doi: 10.1016/j.pt.2015.06.013, PMID: 26433253

[ref21] Bayraktar H. Dolar F. (2009). Genetic diversity of wilt and root rot pathogens of chickpea, as assessed by RAPD and ISSR. Turk. J. Agric. For. 33, 1–10. doi: 10.3906/tar-0709-37

[ref22] Berg L. E. Miller S. S. Dornbusch M. R. Samac D. A. (2017). Seed rot and damping-off of alfalfa in Minnesota caused by *Pythium* and *Fusarium* species. Plant Dis. 101, 1860–1867. doi: 10.1094/PDIS-02-17-0185-RE, PMID: 30677318

[ref23] Bonanomi G. Antignani V. Barile E. Lanzotti V. Scala F. (2011). Decomposition of *Medicago sativa* residues affects phytotoxicity, fungal growth and soil-borne pathogen diseases. J. Plant Pathol. 93, 57–69.

[ref24] Boosalis M. Scharen A. L. (1959). Methods for microscopic detection of *Aphanomyces eutiches* and *Rhizoctonia solani* and for isolation of *Rhizoctonia solani* associated with plant debris. Phytopathology 49, 192–198.

[ref25] Boqvist S. Söderqvist K. Vågsholm I. (2018). Food safety challenges and one health within Europe. Acta Vet. Scand. 60, 1–13. doi: 10.1186/s13028-017-0355-3, PMID: 29298694 PMC5751857

[ref26] Bounou S. Jabaji-Hare S. H. Hogue R. Charest P. M. (1999). Polymerase chain reaction-based assay for specific detection of *Rhizoctonia solani* AG-3 isolates. Mycol. Res. 103, 1–8. doi: 10.1017/S0953756298006522

[ref27] Bradley C. A. Hartman G. L. Wax L. M. Pedersen W. L. (2002). Influence of herbicides on *Rhizoctonia* root and hypocotyl rot of soybean. Crop Prot. 21, 679–687. doi: 10.1016/S0261-2194(02)00021-2

[ref28] Brisbane P. G. Neate S. M. Pankhurst C. E. Scott N. S. Thomas M. R. (1995). Sequence-tagged site markers to identify *Rhizoctonia solani* AG 4 or 8 infecting wheat in South Australia. Phytopathology 85, 1423–1427. doi: 10.1094/Phyto-85-1423

[ref29] Budge G. Shaw M. Colyer A. Pietravalle S. Boonham N. (2009). Molecular tools to investigate *Rhizoctonia solani* distribution in soil. Plant Pathol. 58, 1071–1080. doi: 10.1111/j.1365-3059.2009.02139.x

[ref30] Bulat S. A. Lübeck M. Mironenko N. Jensen D. F. Lübeck P. S. (1998). UP-PCR analysis and ITS1 ribotyping of strains of *Trichoderma* and *Gliocladium*. Mycol. Res. 102, 933–943. doi: 10.1017/S0953756297005686

[ref31] Bustin S. A. (2000). Absolute quantification of mRNA using real-time reverse transcription polymerase chain reaction assays. J. Mol. Endocrinol. 25, 169–193. doi: 10.1677/jme.0.0250169, PMID: 11013345

[ref32] Capote N. Pastrana A. M. Aguado A. Sánchez-Torres P. (2012). Molecular tools for detection of plant pathogenic fungi and fungicide resistance. Plant Pathol. 374, 151–202. doi: 10.5772/38011

[ref33] Carling D. E. Baird R. E. Gitaitis R. D. Brainard K. A. Kuninaga S. (2002). Characterization of AG-13, a newly reported anastomosis group of *Rhizoctonia solani*. Phytopathology 92, 893–899. doi: 10.1094/PHYTO.2002.92.8.893, PMID: 18942969

[ref34] Chang K.-F. Hwang S.-F. Ahmed H. U. Strelkov S. Harding M. Conner R. L. (2017). Disease reaction to *Rhizoctonia solani* and yield losses in soybean. Can. J. Plant Sci. 98, 115–124. doi: 10.1139/CJPS-2017-0053

[ref35] Chavarro-Mesa E. Ceresini P. Pereira D. Vicentini S. Silva T. Ramos-Molina L. (2020). A broad diversity survey of *Rhizoctonia* species from the Brazilian Amazon reveals the prevalence of *R. solani* AG-1 IA on signal grass and the new record of AG-1 IF on cowpea and soybeans. Plant Pathol. 69, 455–466. doi: 10.1111/ppa.13142

[ref36] Chela Fenille R. Luiz de Souza N. Eurya Kuramae E. (2002). Characterization of *Rhizoctonia solani* associated with soybean in Brazil. Eur. J. Plant Pathol. 108, 783–792. doi: 10.1023/A:1020811019189, PMID: 29978936

[ref37] Choudhary P. Rai P. Yadav J. Verma S. Chakdar H. Goswami S. K. (2020). A rapid colorimetric LAMP assay for detection of *Rhizoctonia solani* AG-1 IA causing sheath blight of rice. Sci. Rep. 10, 1–19. doi: 10.1038/s41598-020-79117-0 33328516 PMC7744555

[ref38] Chun S. Gopal J. Muthu M. (2022). A consolidative synopsis of the MALDI-TOF MS accomplishments for the rapid diagnosis of microbial plant disease pathogens. TrAC. 116713.

[ref39] Cong L. Li M. Sun Y. Cong L. Yang Q. Long R. (2016). First report of root rot disease caused by *Fusarium tricinctum* on alfalfa in North China. Plant Dis. 100:1503. doi: 10.1094/PDIS-11-15-1293-PDN

[ref40] Conti M. V. Guzzetti L. Panzeri D. De Giuseppe R. Coccetti P. Labra M. (2021). Bioactive compounds in legumes: implications for sustainable nutrition and health in the elderly population. Trends Food Sci. Technol. 117, 139–147. doi: 10.1016/j.tifs.2021.02.072

[ref41] Contina J.B. (2016). Biological control of *Fusarium solani*, *Rhizoctonia solani* and the pale cyst nematode *Globodera pallida* with Trichoderma harzianum ThzID1. University of Idaho.

[ref42] Cotxarrera L. Trillas-Gay M. Steinberg C. Alabouvette C. (2002). Use of sewage sludge compost and *Trichoderma asperellum* isolates to suppress *Fusarium* wilt of tomato. Soil Biol. Biochem. 34, 467–476. doi: 10.1016/S0038-0717(01)00205-X

[ref43] Cubeta M. A. Vilgalys R. Gonzalez D. (1996). “Molecular analysis of ribosomal RNA genes in *Rhizoctonia* fungi” in Rhizoctonia Species: Taxonomy, Molecular Biology, Ecology, Pathology and Disease Control. eds. Sneh B. Jabaji-Hare S. Neate S. Dijst G. (Dordrecht, Netherland: Springer), 81–86.

[ref44] Das S. Falloon R. E. Stewart A. Pitman A. R. (2016). Novel mitoviruses in *Rhizoctonia solani* AG-3PT infecting potato. Fungal Biol. 120, 338–350. doi: 10.1016/j.funbio.2015.11.002, PMID: 26895862

[ref45] Datema E. Hulzink R. J. Blommers L. Valle-Inclan J. E. Van Orsouw N. Wittenberg A. H. (2016). The megabase-sized fungal genome of *Rhizoctonia solani* assembled from nanopore reads only. BioRxiv 84772

[ref46] de Lannoy C. de Ridder D. Risse J. (2017). The long reads ahead: de novo genome assembly using the MinION. F1000Res. 6, 1–26. doi: 10.12688/f1000research.12012.2 PMC5770995 29375809

[ref47] Demirci E. Eken C. Kantar F. (1998). Wilt and root rot pathogens of chickpea cv.'aziziye-94′. J. Plant Pathol. 80:175.

[ref48] Dorrance A. Kleinhenz M. McClure S. Tuttle N. (2003). Temperature, moisture, and seed treatment effects on *Rhizoctonia solani* root rot of soybean. Plant Dis. 87, 533–538. doi: 10.1094/PDIS.2003.87.5.533, PMID: 30812954

[ref49] Duarte V. Salcedo S. Barreto R. (2018). *Rhizoctonia solani* AG4 causes lentil damping-off in Brazil. Australas. Plant Dis. Notes 13, 1–2. doi: 10.1007/s13314-018-0328-z

[ref50] Dubey S. C. Tripathi A. Upadhyay B. K. Deka U. K. (2014). Diversity of *Rhizoctonia solani* associated with pulse crops in different agro-ecological regions of India. World J. Microbiol. Biotechnol. 30, 1699–1715. doi: 10.1007/s11274-013-1590-z, PMID: 24399024

[ref51] Dubey S. Tripathi A. Upadhyay B. Thakur M. (2011). Pathogenic behaviour of leguminous isolates of *Rhizoctonia solani* collected from different Indian agro-ecological regions. Indian J. Agric. Sci. 81:948.

[ref52] Duncan S. Barton J. E. O'Brien P. A. (1993). Analysis of variation in isolates of *Rhizoctonia solani* by random amplified polymorphic DNA assay. Mycol. Res. 97, 1075–1082. doi: 10.1016/S0953-7562(09)80508-X, PMID: 25062484

[ref53] Duniway J. M. (2002). Status of chemical alternatives to methyl bromide for pre-plant fumigation of soil. Phytopathology 92, 1337–1343. doi: 10.1094/PHYTO.2002.92.12.1337, PMID: 18943890

[ref54] Eken C. Demirci E. (2003). Identification and pathogenicity of *Rhizoctonia solani* and binucleate *Rhizoctonia* anastomosis groups isolated from forage legumes in Erzurum, Turkey. Phytoparasitica 31, 76–80. doi: 10.1007/BF02979769

[ref55] El-Garhy A. M. El-Wakil D. A. (2014). Fungal diseases of alfalfa in Ismailia governorate. Egypt. J. Agric. Res. 92, 1219–1231. doi: 10.21608/ejar.2014.156721

[ref56] El-Meleigi M. Omar A. Rogaibah A. Ibrahim G. (2017). Efficacy of *Bacilli* strains in growth promotion and biological control of soilborne *Rhizoctonia* and *Fusarium* on alfalfa (*Medicago sativa* L.) and potato (*Solanum tuberosum* L). Egypt. J. Agric. Res. 27:85.

[ref57] Erper I. Ozkoc I. Karaca G. H. (2011). Identification and pathogenicity of *Rhizoctonia* species isolated from bean and soybean plants in Samsun, Turkey. Arch. Phytopathol. Plant Protect. 44, 78–84. doi: 10.1080/03235400903395427

[ref58] Ganeshamoorthi P. Dubey S. (2013). Phylogeny analysis of Indian strains of *Rhizoctonia solani* isolated from chickpea and development of sequence characterized amplified region (SCAR) marker for detection of the pathogen. Afr. J. Microbiol. Res. 7, 5516–5525. doi: 10.5897/AJMR2013.5769

[ref59] Gardes M. Bruns T. D. (1993). ITS primers with enhanced specificity for basidiomycetes-application to the identification of mycorrhizae and rusts. Mol. Ecol. 2, 113–118. doi: 10.1111/j.1365-294X.1993.tb00005.x, PMID: 8180733

[ref60] Godoy-Lutz G. Kuninaga S. Steadman J. R. Powers K. (2008). Phylogenetic analysis of *Rhizoctonia solani* subgroups associated with web blight symptoms on common bean based on ITS-5.8 S rDNA. J. Gen. Plant Pathol. 74, 32–40. doi: 10.1007/s10327-007-0060-6

[ref61] Gonzalez D. Carling D. E. Kuninaga S. Vilgalys R. Cubeta M. A. (2001). Ribosomal DNA systematics of *Ceratobasidium* and *Thanatephorus* with *Rhizoctonia* anamorphs. Mycologia 93, 1138–1150. doi: 10.1080/00275514.2001.12063247, PMID: 27020160

[ref62] Gónzalez D. Rodriguez-Carres M. Boekhout T. Stalpers J. Kuramae E. E. Nakatani A. K. (2016). Phylogenetic relationships of *Rhizoctonia* fungi within the *Cantharellales*. Fungal Biol. 120, 603–619. doi: 10.1016/j.funbio.2016.01.012, PMID: 27020160 PMC5013834

[ref63] Graham P. H. Vance C. P. (2003). Legumes: importance and constraints to greater use. Plant Physiol. 131, 872–877. doi: 10.1104/pp.017004, PMID: 12644639 PMC1540286

[ref64] Grosch R. Schneider J. H. M. Peth A. Waschke A. Franken P. Kofoet A. (2007). Development of a specific PCR assay for the detection of *Rhizoctonia solani* AG 1-IB using SCAR primers. J. Appl. Microbiol. 102, 806–819. doi: 10.1111/j.1365-2672.2006.03125.x, PMID: 17309631

[ref65] Guerrero-González M. Rodríguez-Kessler M. Rodríguez-Guerra R. González-Chavira M. Simpson J. Sanchez F. (2011). Differential expression of *Phaseolus vulgaris* genes induced during the interaction with *Rhizoctonia solani*. Plant Cell Rep. 30, 1465–1473. doi: 10.1007/s00299-011-1055-5, PMID: 21416283

[ref66] Hamdi A. Ezzat Z. M. Shaaban M. Shalaby F. Said M. EI-Lathy R. (2002). A new early maturing lentil cultivar: SINA 1. J. Plant Prod. Sci. 27, 3631–3645.

[ref67] Hane J. K. Anderson J. P. Williams A. H. Sperschneider J. Singh K. B. (2014). Genome sequencing and comparative genomics of the broad host-range pathogen *Rhizoctonia solani* AG8. PLoS Genet. 10:e1004281. doi: 10.1371/journal.pgen.1004281, PMID: 24810276 PMC4014442

[ref01] Haggag W. M. Mohamed H. Abdel-Latif A. (2007). Biotechnological aspects of microorganisms used in plant biological control. Am. -Eurasian J. Sustain. Agric. 1, 7–12.

[ref68] Harikrishnan R. Yang X. B. (2004). Recovery of anastomosis groups of *Rhizoctonia solani* from different latitudinal positions and influence of temperatures on their growth and survival. Plant Dis. 88, 817–823. doi: 10.1094/PDIS.2004.88.8.817, PMID: 30812508

[ref69] Heyman F. Blair J. Persson L. Wikström M. (2013). Root rot of pea and faba bean in southern Sweden caused by *Phytophthora pisi* sp. nov. Plant Dis. 97, 461–471. doi: 10.1094/PDIS-09-12-0823-RE, PMID: 30722231

[ref70] Hu J. Vick B. A. (2003). Target region amplification polymorphism: a novel marker technique for plant genotyping. Plant Mol. Biol. Report. 21, 289–294. doi: 10.1007/BF02772804

[ref71] Hwang S. Gossen B. Chang K. Turnbull G. Howard R. Blade S. (2003). Etiology, impact and control of *Rhizoctonia* seedling blight and root rot of chickpea on the Canadian prairies. Can. J. Plant Sci. 83, 959–967. doi: 10.4141/P02-165

[ref72] Hwang S. Gossen B. Conner R. Chang K. Turnbull G. Lopetinsky K. (2007). Management strategies to reduce losses caused by *Rhizoctonia* seedling blight of field pea. Can. J. Plant Sci. 87, 145–155. doi: 10.4141/P04-172

[ref73] Infantino A. Kharrat M. Riccioni L. Coyne C. J. McPhee K. E. Grünwald N. J. (2006). Screening techniques and sources of resistance to root diseases in cool season food legumes. Euphytica 147, 201–221. doi: 10.1007/s10681-006-6963-z

[ref74] Jaaffar A. K. M. Paulitz T. C. Schroeder K. L. Thomashow L. S. Weller D. M. (2016). Molecular characterization, morphological characteristics, virulence, and geographic distribution of *Rhizoctonia* spp. in Washington State. Phytopathology 106, 459–473. doi: 10.1094/PHYTO-09-15-0208-R, PMID: 26780436

[ref75] Jain M. Koren S. Miga K. H. Quick J. Rand A. C. Sasani T. A. (2018). Nanopore sequencing and assembly of a human genome with ultra-long reads. Nat. Biotechnol. 36, 338–345. doi: 10.1038/nbt.4060, PMID: 29431738 PMC5889714

[ref76] Johnk J. S. Jones R. K. (2001). Differentiation of three homogeneous groups of *Rhizoctonia solani* anastomosis group 4 by analysis of fatty acids. Phytopathology 91, 821–830. doi: 10.1094/PHYTO.2001.91.9.821, PMID: 18944227

[ref77] Julián María C. Acero J. Salazar O. Keijer J. Victor Rubio (1999). Mating type-correlated molecular markers and demonstration of heterokaryosis in the phytopathogenic fungus *Thanatephorus cucumeris* (*Rhizoctonia solani*) AG 1-IC by AFLP DNA fingerprinting analysis. J. Biotechnol. 67, 49–56. doi: 10.1016/S0168-1656(98)00167-9, PMID: 9987848

[ref78] Kang Y. J. Kim S. K. Kim M. Y. Lestari P. Kim K. H. Ha B.-K. (2014). Genome sequence of mungbean and insights into evolution within *Vigna* species. Nat. Commun. 5, 1–9. doi: 10.1038/ncomms6443 PMC4241982 25384727

[ref79] Kaufman P. Rothrock C. (1995). Evaluation of isolate diversity of *Rhizoctonia solani* subgroup. Phytopathology 85:1125.

[ref80] Kelly J. D. Gepts P. Miklas P. N. Coyne D. P. (2003). Tagging and mapping of genes and QTL and molecular marker-assisted selection for traits of economic importance in bean and cowpea. Field Crop Res. 82, 135–154. doi: 10.1016/S0378-4290(03)00034-0

[ref81] Keshavarz Tohid V. Taheri P. (2015). Investigating binucleate *Rhizoctonia* induced defence responses in kidney bean against *Rhizoctonia solani*. Biocontrol Sci. Tech. 25, 444–459. doi: 10.1080/09583157.2014.984285

[ref82] Koenning S. R. Wrather J. A. (2010). Suppression of soybean yield potential in the continental United States by plant diseases from 2006 to 2009. Plant Health Prog. 11:5. doi: 10.1094/PHP-2010-1122-01-RS

[ref83] Kraft J. M. Pfleger F. L. (2001). Compendium of pea diseases and pests American Phytopathological Society (St. Paul, USA: APS Press).

[ref84] Kuninaga S. Yokosawa R. (1985). DNA Base sequence homology in *Rhizoctonia solani* Kühn VI. Genetic relatedness among seven anastomosis groups. Japan. J. Phytopathol. 51, 127–132. doi: 10.3186/jjphytopath.51.127

[ref85] Kuramae E. E. Buzeto A. L. Nakatani A. K. Souza N. L. (2007). rDNA-based characterization of a new binucleate *Rhizoctonia* spp. causing root rot on kale in Brazil. Eur. J. Plant Pathol. 119, 469–475. doi: 10.1007/s10658-007-9175-z

[ref86] Kwon Y. S. Kim S. G. Chung W. S. Bae H. Jeong S. W. Shin S. C. (2014). Proteomic analysis of *Rhizoctonia solani* AG-1 sclerotia maturation. Fungal Biol. 118, 433–443. doi: 10.1016/j.funbio.2014.02.001 24863472

[ref87] Lakshman D. K. Jambhulkar P. P. Singh V. Sharma P. Mitra A. (2016). “Molecular identification, genetic diversity, population genetics and genomics of *Rhizoctonia solani*,” in Perspectives of plant pathology in genomic era. eds. Chowdappa P. Sharma P. Singh D. Mitra A. K. (New Delhi, India: Today and Tomorrow’s Printers and Publications), 55–89.

[ref88] Laroche J. Jabaji-Hare S. Charest P. (1992). Differentiation of two anastomosis groups of *Rhizoctonia solani* by isozyme analysis. APS 82, 1387–1387. doi: 10.1094/Phyto-82-1387

[ref89] Lee D.-Y. Jeon J. Kim K.-T. Cheong K. Song H. Choi G. (2021). Comparative genome analyses of four rice-infecting *Rhizoctonia solani* isolates reveal extensive enrichment of homogalacturonan modification genes. BMC Genomics 22, 1–15. doi: 10.1186/s12864-021-07549-7 33827423 PMC8028249

[ref90] Liu Z. L. Domier L. L. Sinclair J. B. (1995). Polymorphism of genes coding for nuclear 18S rRNA indicates genetic distinctiveness of anastomosis group 10 from other groups in the *Rhizoctonia solani* species complex. Appl. Environ. Microbiol. 61, 2659–2664. doi: 10.1128/aem.61.7.2659-2664.1995, PMID: 7618879 PMC167539

[ref91] Liu Y. Hassan S. Kidd B. N. Garg G. Mathesius U. Singh K. B. (2017). Ethylene signaling is important for isoflavonoid-mediated resistance to *Rhizoctonia solani* in roots of *Medicago truncatula*. Mol. Plant-Microbe Interact. 30, 691–700. doi: 10.1094/MPMI-03-17-0057-R, PMID: 28510484

[ref92] Liu S. Proudman J. Mitloehner F. M. (2021a). Rethinking methane from animal agriculture. CABI Agric. Biosci. 2:22. doi: 10.1186/s43170-021-00041-y

[ref93] Liu Y. Rosikiewicz W. Pan Z. Jillette N. Wang P. Taghbalout A. (2021b). DNA methylation-calling tools for Oxford nanopore sequencing: a survey and human epigenome-wide evaluation. Genome Biol. 22, 1–33. doi: 10.1186/s13059-021-02510-z 34663425 PMC8524990

[ref94] López Cardona N. Castaño Zapata J. (2012). Characterization of phytopathogenic fungi, bacteria, nematodes and viruses in four commercial varieties of *Heliconia* (*Heliconia* sp.). Rev. Fac. Nac. Agron. Medellin 65, 6697–6710.

[ref95] Lu C. Song B. Zhang H. Wang Y. Zheng X. (2015). Rapid diagnosis of soybean seedling blight caused by *Rhizoctonia solani* and soybean charcoal rot caused by *Macrophomina phaseolina* using LAMP assays. Phytopathology 105, 1612–1617. doi: 10.1094/PHYTO-01-15-0023-R, PMID: 26606587

[ref96] Lübeck M. (2004). “Molecular characterization of *Rhizoctonia solani*” in Applied mycology and biotechnology. Volume 4: fungal genomics. eds. Arora D. K. Khachatourians G. G. (Amsterdam, Netherlands: Elsevier Science B.V.), 205–224.

[ref97] Lübeck M. Lübeck P. S. (2005). “Universally primed PCR (UP-PCR) and its applications in mycology” in Biodiversity of fungi: their role in human life. eds. Deshmukh S. K. Rai M. K. (Enfield, USA: Science Publishers, Inc.), 409–438.

[ref98] Lübeck M. Poulsen H. (2001). UP-PCR cross blot hybridization as a tool for identification of anastomosis groups in the *Rhizoctonia solani* complex. FEMS Microbiol. Lett. 201, 83–89. doi: 10.1016/S0378-1097(01)00245-2, PMID: 11445172

[ref99] Mahmoud Y. A. Gaafar R. M. Mubarak H. (2007). Genetic diversity among Nile Delta isolates of *Rhizoctonia solani* Kühn based on pathogenicity, compatibility, isozyme analysis and total protein pattern. Turk. J. Bot. 31, 19–29.

[ref100] Mahmud K. Makaju S. Ibrahim R. Missaoui A. (2020). Current progress in nitrogen fixing plants and microbiome research. Plan. Theory 9:97. doi: 10.3390/plants9010097, PMID: 31940996 PMC7020401

[ref101] Marsalis M.A. Puppala N. Goldberg N.P. Ashigh J. Sanogo S. Trostle C. (2009). New Mexico peanut production. Cooperative Extension Service, College of Agricultural, Consumer and Environmental Sciences, 2009.

[ref102] Martinson C. (1963). Inoculum potential relationships of *Rhizoctonia solani* measured with soil microbiological sampling tubes. Phytopathology 53:634.

[ref103] Mat Razali N. Hisham S. N. Kumar I. S. Shukla R. N. Lee M. Abu Bakar M. F. (2021). Comparative genomics: insights on the pathogenicity and lifestyle of *Rhizoctonia solani*. Int. J. Mol. Sci. 22:2183. doi: 10.3390/ijms22042183, PMID: 33671736 PMC7926851

[ref104] Mathew F. Lamppa R. Chittem K. Chang Y. Botschner M. Kinzer K. (2012). Characterization and pathogenicity of *Rhizoctonia solani* isolates affecting *Pisum sativum* in North Dakota. Plant Dis. 96, 666–672. doi: 10.1094/PDIS-02-11-0087, PMID: 30727512

[ref105] Maurice S. M. Kamel K. S. Moawad R. O. Amal A. A. Khaled K. K. (2010). Current *Rhizoctonia solani* anastomosis groups in Egypt and their pathogenic relation to cotton seedlings. Afr. J. Microbiol. Res. 4, 386–395.

[ref106] Mayo S. Gutierrez S. Malmierca M. G. Lorenzana A. Campelo M. P. Hermosa R. (2015). Influence of *Rhizoctonia solani* and *Trichoderma* spp. in growth of bean (*Phaseolus vulgaris* L.) and in the induction of plant defense-related genes. Front. Plant Sci. 6:685. doi: 10.3389/fpls.2015.00685, PMID: 26442006 PMC4584982

[ref107] McCartney H. A. Foster S. J. Fraaije B. A. Ward E. (2003). Molecular diagnostics for fungal plant pathogens. Pest Manag. Sci. 59, 129–142. doi: 10.1002/ps.575, PMID: 12587866

[ref108] Misawa T. Kurose D. (2019). Anastomosis group and subgroup identification of *Rhizoctonia solani* strains deposited in NARO Genebank. Japan. J. Gen. Plant Pathol. 85, 282–294. doi: 10.1007/s10327-019-00848-8

[ref109] Misawa T. Kurose D. Mori M. Toda T. (2018). Characterization of Japanese *Rhizoctonia solani* AG-2-1 isolates using rDNA-ITS sequences, culture morphology, and growth temperature. J. Gen. Plant Pathol. 84, 387–394. doi: 10.1007/s10327-018-0808-1

[ref110] Misawa T. Kurose D. Shishido K. Toda T. Kuninaga S. (2020). Characterization of a new subgroup of *Rhizoctonia solani* anastomosis group 3 (AG-3 TM) associated with tomato leaf blight. J. Gen. Plant Pathol. 86, 457–467. doi: 10.1007/s10327-020-00943-1

[ref111] Montesinos E. (2003). Development, registration and commercialization of microbial pesticides for plant protection. Int. Microbiol. 6, 245–252. doi: 10.1007/s10123-003-0144-x, PMID: 12955583

[ref112] Mueller-Harvey I. Bee G. Dohme-Meier F. Hoste H. Karonen M. Kölliker R. (2019). Benefits of condensed tannins in forage legumes fed to ruminants: importance of structure, concentration, and diet composition. Crop Sci. 59, 861–885. doi: 10.2135/cropsci2017.06.0369

[ref113] Muyolo N. Lipps P. Schmitthenner A. (1993). Reactions of dry bean, lima bean, and soybean cultivars to *Rhizoctonia* root and hypocotyl rot and web blight. Plant Dis. 77, 234–238. doi: 10.1094/PD-77-0234

[ref114] Narayanasamy P. (ed.) (2011). “Assessment of variability in plant viral and viroid pathogens” in Microbial plant pathogens-detection and disease: diagnosis. (Dordrecht, Netherlands: Springer), 251–294.

[ref115] Nene Y. Reddy M. Haware M. Ghanekar A. Amin K. Pande S. (2012). Field diagnosis of chickpea diseases and their control. Information bulletin no. 28 (revised). International crops research Institute for the Semi-Arid Tropics.

[ref116] Noble R. Coventry E. (2005). Suppression of soil-borne plant diseases with composts: a review. Biocontrol Sci. Tech. 15, 3–20. doi: 10.1080/09583150400015904

[ref117] Notomi T. Okayama H. Masubuchi H. Yonekawa T. Watanabe K. Amino N. (2000). Loop-mediated isothermal amplification of DNA. Nucleic Acids Res. 28, e63–e63. 10871386 10.1093/nar/28.12.e63 PMC102748

[ref118] O’Briert P. A. Zamani M. (2003). Production of pectic enzymes by barepatch isolates of *Rhizoctonia solani* AG 8. Australas. Plant Pathol. 32, 65–72. doi: 10.1071/AP02073

[ref119] Ogoshi A. (1987). Ecology and pathogenicity of anastomosis and intraspecific groups of *Rhizoctonia solani* Kuhn. Annu. Rev. Phytopathol. 25, 125–143. doi: 10.1146/annurev.py.25.090187.001013

[ref120] Ogoshi A. (1996). “Introduction—The genus *Rhizoctonia*,” in Rhizoctonia Species: Taxonomy, Molecular Biology, Ecology, Pathology and Disease Control. eds. Sneh B. Jabaji-Hare S. Neate S. Dijst G. (Dordrecht, Netherland: Springer), 1–9.

[ref121] Oladzad A. Zitnick-Anderson K. Jain S. Simons K. Osorno J. M. McClean P. E. (2019). Genotypes and genomic regions associated with *Rhizoctonia solani* resistance in common bean. Front. Plant Sci. 10:956. doi: 10.3389/fpls.2019.00956 31396253 PMC6667560

[ref122] Oliveira A. C. S. Rosa M. C. Jéssica L. B. Yasmine A. M. Milena M. A. F. Gabrielle Virgínia F. C. (2018). Validating the efficiency of a simplex PCR and quantitative SYBR green qPCR for the identification of *Salmonella* spp. J. Food Microbiol. Saf. Hyg. 3, 2476–2059. doi: 10.4172/2476-2059.1000130

[ref123] Ozbay N. Newman S. E. (2004). Biological control with *Trichoderma* spp. with emphasis on *T. harzianum*. Pak. J. Biol. Sci. 7, 478–484.

[ref124] Pane C. Spaccini R. Piccolo A. Scala F. Bonanomi G. (2011). Compost amendments enhance peat suppressiveness to *Pythium ultimum*, *Rhizoctonia solani* and *Sclerotinia minor*. Biol. Control 56, 115–124. doi: 10.1016/j.biocontrol.2010.10.002

[ref125] Pannecoucque J. Hofte M. (2009). Detection of rDNA ITS polymorphism in *Rhizoctonia solani* AG 2-1 isolates. Mycologia 101, 26–33. doi: 10.3852/08-084 19271668

[ref126] Papavizas G. Davey C. (1959). Isolation of *Rhizoctonia solani* Kuehn from naturally infested and artificially inoculated soils. Plant Dis. Rep. 43, 404–410.

[ref127] Park H.-J. Kim S.-H. Kim H.-J. Choi S.-H. (2006). A new composition of nanosized silica-silver for control of various plant diseases. Plant Pathol. J. 22, 295–302. doi: 10.5423/PPJ.2006.22.3.295

[ref128] Pascual J. Hernandez T. Garcia C. De Leij F. Lynch J. (2000). Long-term suppression of *Pythium ultimum* in arid soil using fresh and composted municipal wastes. Biol. Fertil. Soils 30, 478–484.

[ref129] Patel J. S. Brennan M. S. Khan A. Ali G. S. (2015). Implementation of loop-mediated isothermal amplification methods in lateral flow devices for the detection of *Rhizoctonia solani*. Can. J. Plant Pathol. 37, 118–129. doi: 10.1080/07060661.2014.996610

[ref130] Patil H. J. Solanki M. K. (2016). “Molecular prospecting: advancement in diagnosis and control of *Rhizoctonia solani* diseases in plants,” in Current Trends in Plant Disease Diagnostics and Management Practices. eds. Kumar P. Gupta V. Tiwari A. Kamle M. (Cham, Switzerland: Springer), 165–185.

[ref131] Paulitz T. Nowak-Thompson B. Gamard P. Tsang E. Loper J. (2000). A novel antifungal furanone from *Pseudomonas aureofaciens*, a biocontrol agent of fungal plant pathogens. J. Chem. Ecol. 26, 1515–1524. doi: 10.1023/A:1005595927521

[ref132] Paulitz T. Schroeder K. (2005). A new method for the quantification of *Rhizoctonia solani* and *R. oryzae* from soil. Plant Dis. 89, 767–772. doi: 10.1094/PD-89-0767, PMID: 30791249

[ref133] Poore J. Nemecek T. (2018). Reducing food’s environmental impacts through producers and consumers. Science 360, 987–992. doi: 10.1126/science.aaq0216, PMID: 29853680

[ref134] Prasad J. Gaur V. Mehta S. (2014). Pathogenicity and characterization of *Rhizoctonia solani* Kühn inciting wet root rot in chickpea. J. Rural Agric. Res. 14, 12–14.

[ref135] Rehman H. M. Cooper J. W. Lam H. M. Yang S. H. (2019). Legume biofortification is an underexploited strategy for combatting hidden hunger. Plant Cell Environ. 42, 52–70. doi: 10.1111/pce.13368, PMID: 29920691

[ref136] Rivera M. Wright E. Lopez M. Garda D. Barrague M. (2004). Promotion of growth and control of damping-off (*Rhizoctonia solani*) of greenhouse tomatoes amended with vermicompost. Phyton 73, 229–235.

[ref137] Röös E. de Groote A. Stephan A. (2022). Meat tastes good, legumes are healthy and meat substitutes are still strange - the practice of protein consumption among Swedish consumers. Appetite 174:106002. doi: 10.1016/j.appet.2022.106002, PMID: 35341881

[ref138] Salazar O. Julián M. C. Hyakumachi M. Rubio V. (2000). Phylogenetic grouping of cultural types of *Rhizoctonia solani* AG 2–2 based on ribosomal ITS sequences. Mycologia 92, 505–509.

[ref139] Samac D. A. Schraber S. Barclay S. (2015). A mineral seed coating for control of seedling diseases of alfalfa suitable for organic production systems. Plant Dis. 99, 614–620. doi: 10.1094/PDIS-03-14-0240-RE, PMID: 30699682

[ref140] Sharma-Poudyal D. Paulitz T. C. Porter L. D. du Toit L. J. (2015). Characterization and pathogenicity of *Rhizoctonia* and *Rhizoctonia*-like spp. from pea crops in the Columbia basin of Oregon and Washington. Plant Dis. 99, 604–613. doi: 10.1094/PDIS-08-14-0803-RE, PMID: 30699678

[ref141] Sharon M. Freeman S. Sneh B. (2011). Assessment of resistance pathways induced in *Arabidopsis thaliana* by hypovirulent *Rhizoctonia* spp. isolates. Phytopathology 101, 828–838. doi: 10.1094/PHYTO-09-10-0247, PMID: 21385012

[ref142] Sharon M. Sneh B. Kuninaga S. Hyakumachi M. Naito S. (2008). Classification of *Rhizoctonia* spp. using rDNA-ITS sequence analysis supports the genetic basis of the classical anastomosis grouping. Mycoscience 49, 93–114. doi: 10.1007/S10267-007-0394-0

[ref143] Simpson J. T. Workman R. Zuzarte P. C. David M. Dursi L. J. Timp W. (2016). Detecting DNA methylation using the oxford nanopore technologies MinION sequencer. BioRxiv 47142

[ref144] Sneh B. Jabaji-Hare S. Neate S. Dijst G. (1996). Rhizoctonia species: Taxonomy, molecular biology, ecology, pathology and disease control. Dordrecht, Netherlands: Springer.

[ref0110] Spadaro D. Gullino M. L. (2014). State of the art and future prospects of the biological control of postharvest fruit diseases. Int. J. Food Microbiol. 91, 185–194. doi: 10.1016/S0168-1605(03)00380-5 14996462

[ref145] Spedaletti Y. Aparicio M. Mercado Cardenas G. Rodriguero M. Taboada G. Aban C. (2016). Genetic characterization and pathogenicity of *Rhizoctonia solani* associated with common bean web blight in the main bean growing area of Argentina. J. Phytopathol. 164, 1054–1063. doi: 10.1111/jph.12526

[ref146] Spurlock T. N. Rothrock C. S. Monfort W. S. Griffin T. W. (2016). The distribution and colonization of soybean by *Rhizoctonia solani* AG11 in fields rotated with rice. Soil Biol. Biochem. 94, 29–36. doi: 10.1016/j.soilbio.2015.11.002

[ref147] Stodart B. J. Harvey P. R. Neate S. M. Melanson D. L. Scott E. S. (2007). Genetic variation and pathogenicity of anastomosis group 2 isolates of *Rhizoctonia solani* in Australia. Mycol. Res. 111, 891–900. doi: 10.1016/j.mycres.2007.05.008, PMID: 17707626

[ref148] Thiessen L.D. Woodward J.E. (2012). Diseases of peanut caused by soilborne pathogens in the southwestern United States. International scholarly research notices 2012.

[ref149] Thornton C. Dewey F. Gilligan C. (1993). Development of monoclonal antibody-based immunological assays for the detection of live propagules of *Rhizoctonia solani* in soil. Plant Pathol. 42, 763–773. doi: 10.1111/j.1365-3059.1993.tb01563.x

[ref150] Tilman D. Cassman K. G. Matson P. A. Naylor R. Polasky S. (2002). Agricultural sustainability and intensive production practices. Nature 418, 671–677. doi: 10.1038/nature01014, PMID: 12167873

[ref151] Tomlinson J. Dickinson M. Boonham N. (2010). Rapid detection of *Phytophthora ramorum* and *P. kernoviae* by two-minute DNA extraction followed by isothermal amplification and amplicon detection by generic lateral flow device. Phytopathology 100, 143–149. doi: 10.1094/PHYTO-100-2-0143, PMID: 20055648

[ref152] United Nations (2017). World population prospects. Geneva 1211, Switzerland: Multimedia Library.

[ref153] Vågsholm I. Arzoomand N. S. Boqvist S. (2020). Food security, safety, and sustainability—getting the trade-offs right. Front. Sustain. Food Syst. 4:16. doi: 10.3389/fsufs.2020.00016, PMID: 35561496

[ref154] Valentín Torres S. Vargas M. M. Godoy-Lutz G. Porch T. G. Beaver J. S. (2016). Isolates of *Rhizoctonia solani* can produce both web blight and root rot symptoms in common bean (*Phaseolus vulgaris* L.). Plant Dis. 100, 1351–1357. doi: 10.1094/PDIS-11-15-1270-RE, PMID: 30686205

[ref155] Vilgalys R. Gonzalez D. (1990). Organization of ribosomal DNA in the basidiomycete *Thanatephorus praticola*. Curr. Genet. 18, 277–280. doi: 10.1007/BF00318394, PMID: 2249259

[ref156] Wen K. Seguin P. St. Arnaud M. Jabaji-Hare S. (2005). Real-time quantitative RT-PCR of defense-associated gene transcripts of *Rhizoctonia solani*-infected bean seedlings in response to inoculation with a nonpathogenic binucleate *Rhizoctonia* isolate. Phytopathology 95, 345–353. doi: 10.1094/PHYTO-95-0345, PMID: 18943035

[ref157] Whisson D. L. Francis L. (1995). Detection of *Rhizoctonia solani* AG-8 in soil using a specific DNA probe. Mycol. Res. 99, 1299–1302. doi: 10.1016/S0953-7562(09)81211-2

[ref158] Wibberg D. Andersson L. Rupp O. Goesmann A. Pühler A. Varrelmann M. (2016). Draft genome sequence of the sugar beet pathogen *Rhizoctonia solani* AG2-2IIIB strain BBA69670. J. Biotechnol. 222, 11–12. doi: 10.1016/j.jbiotec.2016.02.001, PMID: 26851388

[ref159] Widmer T. Graham J. Mitchell D. (1999). Composted municipal solid wastes promote growth of young citrus trees infested with *Phytophthora nicotianae*. Compost Sci. Util. 7, 6–16. doi: 10.1080/1065657X.1999.10701959

[ref160] Willett W. Rockström J. Loken B. Springmann M. Lang T. Vermeulen S. (2019). Food in the Anthropocene: the EAT–lancet commission on healthy diets from sustainable food systems. Lancet 393, 447–492. doi: 10.1016/S0140-6736(18)31788-4, PMID: 30660336

[ref161] Woodhall J. Oropeza A. Guggenheim R. Keith S. Wharton P. (2019). First report of *Rhizoctonia solani* AG4 HG-II causing stem rot of snow pea in Idaho. Plant Dis. 103, 2668–2668. doi: 10.1094/PDIS-05-19-0990-PDN

[ref162] Xia Y. Fei B. He J. Zhou M. Zhang D. Pan L. (2017). Transcriptome analysis reveals the host selection fitness mechanisms of the *Rhizoctonia solani* AG1IA pathogen. Sci. Rep. 7, 1–16. doi: 10.1038/s41598-017-10804-1 28860554 PMC5579035

[ref163] Yamamoto N. Wang Y. Lin R. Liang Y. Liu Y. Zhu J. (2019). Integrative transcriptome analysis discloses the molecular basis of a heterogeneous fungal phytopathogen complex, *Rhizoctonia solani* AG-1 subgroups. Sci. Rep. 9, 1–14. doi: 10.1038/s41598-019-55734-2 31873088 PMC6928066

[ref164] Yang G. Chen H. Naito S. Ogoshi A. Deng Y. (2005). First report of AG-A of binucleate *Rhizoctonia* in China, pathogenic to soya bean, pea, snap bean and pak choy. J. Phytopathol. 153, 333–336. doi: 10.1111/j.1439-0434.2005.00980.x, PMID: 30727498

[ref165] Yimer S. M. Ahmed S. Fininsa C. Tadesse N. Hamwieh A. Cook D. R. (2018). Distribution and factors influencing chickpea wilt and root rot epidemics in Ethiopia. Crop Prot. 106, 150–155. doi: 10.1016/j.cropro.2017.12.027

[ref166] Yobo K. Laing M. Hunter C. (2010). Application of selected biological control agents in conjunction with tolclofos-methyl for the control of damping-off caused by *Rhizoctonia solani*. Afr. J. Biotechnol. 9, 1789–1796. doi: 10.5897/AJB10.1171

[ref167] Youssef N. O. B. Krid S. Rhouma A. Kharrat M. (2010). First report of *Rhizoctonia solani* AG 2-3 on chickpea in Tunisia. Phytopathol. Mediterr. 49, 253–257.

[ref168] Zhao G. Ablett G. Anderson T. Rajcan I. Schaafsma A. (2005). Anastomosis groups of *Rhizoctonia solani* associated with soybean root and hypocotyl rot in Ontario and resistance of accession PI 442031 to different anastomosis groups. Can. J. Plant Pathol. 27, 108–117. doi: 10.1080/07060660509507201

[ref169] Zheng A. Lin R. Zhang D. Qin P. Xu L. Ai P. (2013). The evolution and pathogenic mechanisms of the rice sheath blight pathogen. Nat. Commun. 4, 1–10. doi: 10.1038/ncomms2427 PMC3562461 23361014

